# Candidates for Balancing Selection in *Leishmania donovani* Complex Parasites

**DOI:** 10.1093/gbe/evab265

**Published:** 2021-12-02

**Authors:** Cooper Alastair Grace, Sarah Forrester, Vladimir Costa Silva, Kátia Silene Sousa Carvalho, Hannah Kilford, Yen Peng Chew, Sally James, Dorcas L Costa, Jeremy C Mottram, Carlos C H N Costa, Daniel C Jeffares

**Affiliations:** 1 Department of Biology, York Biomedical Research Institute, University of York, York, United Kingdom; 2 Instituto de Doenças do Sertão, Instituto de Doenças Tropicais Natan Portella, Centro de Ciências da Saúde da Universidade Federal do Piauí, Teresina-PI, Brazil; 3 Institute of Molecular Plant Sciences, University of Edinburgh, Edinburgh, United Kingdom

**Keywords:** *Leishmania*, parasites, balancing selection, genomes, evolution

## Abstract

The *Leishmania donovani* species complex is the causative agent of visceral leishmaniasis, which cause 20–40,000 fatalities a year. Here, we conduct a screen for balancing selection in this species complex. We used 384 publicly available *L. donovani* and *L. infantum* genomes, and sequence 93 isolates of *L. infantum* from Brazil to describe the global diversity of this species complex. We identify five genetically distinct populations that are sufficiently represented by genomic data to search for signatures of selection. We find that signals of balancing selection are generally not shared between populations, consistent with transient adaptive events, rather than long-term balancing selection. We then apply multiple diversity metrics to identify candidate genes with robust signatures of balancing selection, identifying a curated set of 24 genes with robust signatures. These include zeta toxin, nodulin-like, and flagellum attachment proteins. This study highlights the extent of genetic divergence between *L. donovani* complex parasites and provides genes for further study.

Significance

Protozoan parasites of the *Leishmania donovani* species complex are globally distributed, with major foci in East Africa, the Indian subcontinent and Brazil. Although global genetic diversity has been described, there has been very little focus on selective pressures in this species. We used a data set of 477 sequenced isolates to search for signals of balancing selection in populations covering these foci, identifying 24 genes with robust signatures of balancing selection. By identifying genes that appear to be subject to strong selection this study contributes to our understanding of the genetic diversity in this parasite, and parasites in general.

## Introduction

Intracellular *Leishmania* parasites cause the neglected infectious disease leishmaniasis in over 80 countries. Visceral leishmaniasis (VL) is the most severe form of the disease, caused by *Leishmania donovani* and *Leishmania infantum*. Annual cases of VL are estimated at a minimum of 50,000, with a fatality of ≥95% if untreated, and occur primarily in the Indian subcontinent (ISC), Bangladesh, Sudan, South Sudan, Ethiopia, and Brazil (World Health Organisation 2020). After transmission by sand flies, *Leishmania* promastigotes are taken up by macrophages and develop into amastigotes which proliferate. These processes require specific adaptations to different environments, such as evasion and active modulation of mammalian host or sand fly vector immune responses (Atayde et al. 2016; Dong et al. 2019). *Leishmania* species contain genomes that are primarily diploid and sexually recombining. Amongst their unusual features are the use of constitutively transcribed polycistronic genes and supernumerary chromosomes with unstable ploidy (Dumetz et al. 2017).

Balancing selection (BS) has been studied in *Plasmodium* parasites extensively. In this genus, proteins that interact directly with host cells maintain high genetic diversity (Mobegi et al. 2014; Ochola-Oyier et al. 2019; Hocking et al. 2020), as do proteins that are exported to the surface of erythrocytes (Jeffares et al. 2007). BS signals are also enriched in solvent-exposed regions of proteins consistent with selection for increased diversity via a rare allele advantage (Guy et al. 2018). Given the competitive interaction between *Leishmania* cells and host immune cells (Atayde et al. 2016; Dong et al. 2019), BS may also operate in this parasite if rare (parasite) alleles provide an advantage to host–parasite interactions. Other mechanisms of BS, such as heterozygote advantage (overdominance) or alleles that confer fitness differentially in the sand fly vector and the mammal host are also possible. These processes are expected to generate similar genetic signals (Charlesworth 2006). In all these scenarios, genomic signatures of BS can highlight genes that are important for transmission, host immune evasion, or ecological adaptation.

Thus far, there have been no published studies of BS in *Leishmania* species. Here, we use genome data from 477 clinical isolates from the *L. donovani* species complex (*L. infantum* or *L. donovani*) from East Africa, the ISC, and Brazil to identify five populations that are well-represented by genome data. Using a variety of metrics, we search for signals of BS within these populations. We identify multiple strong signatures of BS. Signatures are generally unique to a single population consistent with adaptive divergence between populations.

## Results

### *Leishmania donovani* Complex Genome Data and Population Structure

**Fig. 1.**
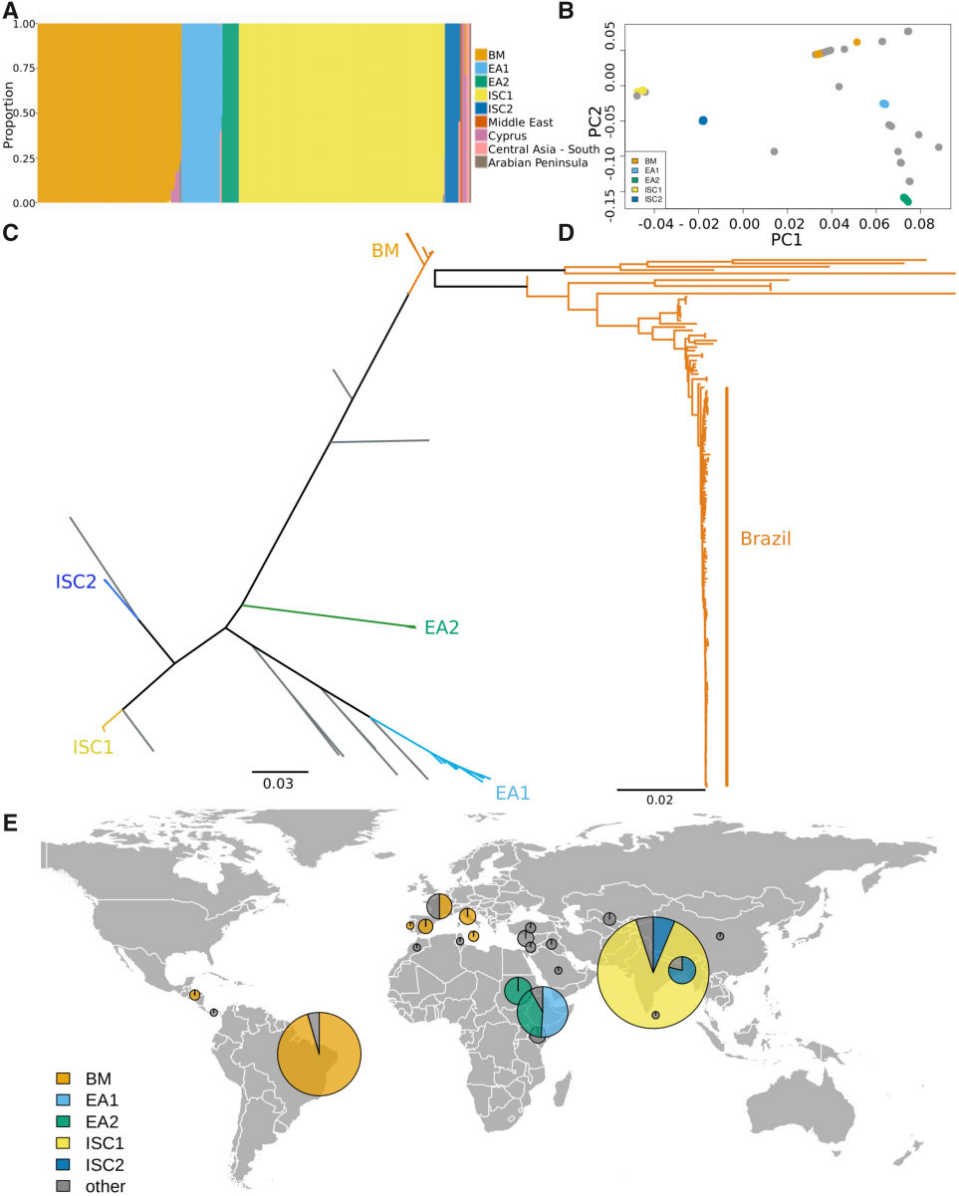
Population structure of the *Leishmania donovani* complex. (*A*) ADMIXTURE analysis indicated between 8 and 11 populations, here *K* = 9. Crossvalidation error values are available in supplementary figure 1, Supplementary Material online. ADMIXTURE plots for *K* = 8, 10 and 11 populations available in supplementary figure 2, Supplementary Material online. (*B*) Principal component analysis (PCA). Strains are colored as for (*A*). Isolates in gray were not confidently assigned to one of the five major populations (BM, EA1, EA2, ISC1, and ISC2) by ADMIXTURE. (*C*) Unrooted ML phylogeny, based upon an SNP alignment of 477 sequences with 283,378 variable sites. All visible branches are maximally supported (100% mlBP) unless indicated. The scale bar represents the number of nucleotide changes per site. Country names in gray indicate origins of isolates that were not confidently assigned to one of the five major populations. (*D*) BM ML tree of *L. infantum* strains, based upon an SNP alignment of 158 sequences with 81,018 variable sites, midpoint rooted. Ninety-three of these isolates were sequenced in the current work. Scale bar and support are as in (*C*). A version of this tree with all isolate origins is available in supplementary figure 3, Supplementary Material online. A single sample isolated in China (Franssen et al. 2020), is the only demographic exception in the BM sample collection, and is indicative of movement of parasites. Data and tree files are available in supplementary documents, Supplementary Material online. (*E*) Locations of samples used in this study. Pie charts show the number of samples from each location that are confidently assigned to one of the five major populations, with a radius proportional to the number of samples from each location. Gray indicates isolates that were not confidently assigned to one of the five major populations.

In this study we used population-scale genomic data from *L. donovani* species complex covering the main global foci of East Africa, the ISC, Brazil, and Europe. We utilized 229 *L. donovani* isolates from the ISC (Imamura et al. 2016), 43 *L. donovani* isolates from Ethiopia (Zackay et al. 2018), 25 *L. infantum* isolates from Brazil (Carnielli et al. 2018), and 87 *L. donovani* isolates from a variety of locations including Sudan (14 strains), France (6), and Israel (10) (Franssen et al. 2020). Additionally, we sequenced 93 *L. infantum* isolates from Piauí state, Brazil (fig. 1, supplementary table 1, Supplementary Material online). This produced a data set of 477 sequenced isolates from the *L. donovani* complex, expanding on the recent analysis of Franssen et al. (2020). To detect genetic variants in these genomes we mapped reads from all isolates to the *L. donovani* BPK282A1 reference genome, and applied variant calling methods and filtering to identify single-nucleotide polymorphisms (SNPs) and insertion/deletion polymorphisms (indels). In this data set of 477 isolates, we identify 339,367 SNPs and 14,383 indels.

We used the ADMIXTURE clustering tool (Alexander and Lange 2011) to assign isolates to populations. This analysis indicated that this collection can be clustered into between 8 and 11 populations (fig. 1; supplementary figs. 1 and 2, Supplementary Material online). The majority of these isolates could be assigned with ≥99% confidence to one of five relatively well-sampled populations (fig. 1). Principal component and phylogenetic analysis showed consistent results. These five populations included two from the Indian subcontinent (ISC1, ISC2), two from East Africa (EA2 from North Ethiopia/Sudan, and EA1 which corresponds to a population from South Ethiopia/Kenya; Gelanew 2010) and a Brazil-Mediterranean population (BM; also contains isolates from Honduras and Panama but we refer to this population as BM from here onwards for brevity). The remaining isolates were assigned to populations of <6 isolates (*n* = 44).

Our results are generally consistent with previous analysis (Franssen et al. 2020), indicating that these five populations have largely independent ancestries. Fixation index (*F*_ST_) values range from 0.27 to 0.90 (supplementary table 2, Supplementary Material online). As has been observed previously (Gelanew 2010), the two East African populations and the older ISC population (ISC2) are approximately equidistant from one another, with *F*_ST_ in the range of ∼0.3. Larger *F*_ST_ values appear to be due to genetic divergence from the newly emerged ISC1 and BM populations. Only 7% of polymorphic sites are shared between two or more populations. We note that rare hybrids have been shown to occur between *L. donovani* complex populations in both East Africa and Turkey (Rogers et al. 2014; Cotton et al. 2020). We do not include *L. donovani* complex hybrids from Turkey (Rogers et al. 2014) in our analysis, because hybrid populations may contain balanced alleles from the parental populations that give an appearance of BS. This, and the under-sampling of VL-endemic regions between Europe and India, render our data unsuited to studying the true extent of global gene flow in this species, so we do not analyze this further here.

**Fig. 2.**
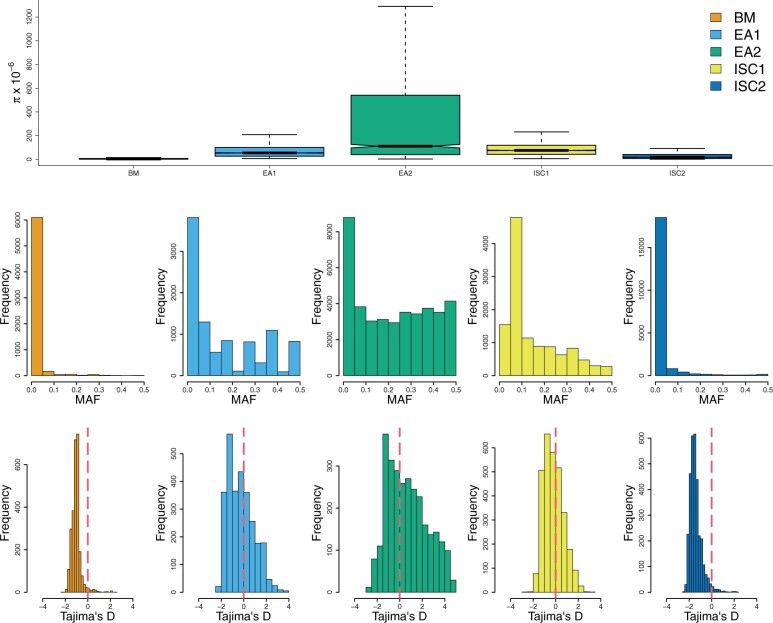
Population genetic statistics. *Upper panel*: nucleotide diversity (π) × 10^−6^, with the box upper and lower limits corresponding to the upper and lower quartiles of π calculated in 10 kb windows; *middle panel*: minor allele frequency (MAF); *lower panel*: Tajima’s *D*.

Phylogenetic analysis provides some qualitative insight to the history of these species (fig. 1*C* and *D*). The long-branched positions of EA1 and EA2 support the relative age of these populations in East Africa, as does the high genetic diversity in this region and genetic distance between these populations, consistent with previous studies (Gelanew et al. 2010, 2014; Ferreira et al. 2012; Teixeira et al. 2017; Zackay et al. 2018; Cotton et al. 2020; Franssen et al. 2020). The high nucleotide diversity of EA1 (table 1) is reflected in the branch lengths in this clade of the phylogeny. In contrast, the smaller ISC population, identified as ISC1 here (equivalent to the ISC5 group identified by Imamura et al. [2016]), produces short terminal branches in the phylogeny and lower genetic diversity (table 1 and fig. 2), consistent with previous genomic analyses indicating that is an emergent population (Imamura et al. 2016). Epidemiological evidence indicates that this population arose in the 1970s after the malaria elimination program (Dye and Wolpert 1988; Bhattacharya et al. 2006; Thakur 2007; Muniaraj 2014; Dhiman and Yadav 2016). The 93 Brazilian isolates we examined, which are mostly from Piauí state in north west Brazil, cluster within isolates originating from Mediterranean countries (fig. 1*D*), consistent with a relatively recent European introduction of *L. infantum* into Brazil (<400 years ago; Kuhls et al. 2011). Short branches in the Brazilian clade (fig. 1*C* and *D*), low-genetic diversity and an abundance of rare alleles (fig. 2) are all consistent with a previously occurring population bottleneck and an expanding population (i.e., a founder effect caused by the transportation of *L. infantum* to Brazil). In contrast, both East African populations and the older population from the ISC2 show higher genetic diversity, and are likely to have been maintained as larger populations for longer periods of time.

**Table 1:** Population Statistics for *Leishmania donovani* Complex Populations

Population	Source	No. of Isolates	No. of Nonadmixed Isolatesa	No. of Private SNPs	No. of Private Indels	**Nucleotide Diversity (π** × **10^−6^)**	Tajima’s *D*	**Mean Minor Allele Frequency (MAF)** ^b^
EA1	East Africa	41	41	3,033	705	424	0.70	0.23
EA2	East Africa	18	18	970	575	87	−0.20	0.14
ISC1	India	225	211	2,689	551	84	−0.23	0.17
ISC2	India	15	15	3,103	716	6.3	−1.04	0.009
BM	Brazil, Med.	133^c^	127	8,886	1,578	28	−1.42	0.02

Note.—Tajima’s *D* and π are mean values for all 10-kb genome windows, calculated within each population.

a Isolates determined as nonadmixed by ADMIXTURE analysis in figure 1.

b Mean MAF is the mean minor allele frequency for all SNPs and indels, calculated across 10 kb windows of all variants, within each population.

c Ninety-three isolates sequenced in this study.

As well as the very low diversity, the BM and smaller East African (EA2) populations contain more indel polymorphisms (table 1), with an SNP:indel ratio of 6:1 and 5:1, respectively, compared with EA1 (11:1), ISC1 (10:1), and ISC2 (9:1), as expected for eukaryote polymorphisms, where SNPs typically outnumber indels (Mullaney et al. 2010; Jeffares et al. 2015). Extensive variant-calling quality control showed that this excess of indels is unlikely to be artefactual (supplementary fig. 4, Supplementary Material online). It is possible that this is due to the accumulation of weakly deleterious indel alleles when the Brazilian population was established from European *L. infantum* populations (Boité et al. 2019).

### Analysis of *L. donovani* Species Complex Populations

Genetic diversity summary statistics vary considerably between populations (fig. 2 and table 1), consistent with these varied demographic histories in different locations. For example, the initial population within the ISC (ISC1) has a normally distributed Tajimas’s *D*, whereas the Tajimas’s *D* is strongly skewed to negative values in the emerging ISC2 population. To select groups of strains that will approximate panmictic populations, we used the ADMIXTURE analysis to identify isolates that were confidently assigned to one population (*n* = 433), rather than being interpopulation hybrids. This selection resulted in 59 isolates from East Africa (two populations of 41 and 18), 226 from the ISC (two populations of 211 and 15), and 127 from BM, of which 93 were newly sequenced here (table 1). Our assignment of isolates into five major populations largely agrees with the previous global analysis of Franssen et al (2020). To characterize BS in these populations we applied the *NCD2* test (Bitarello et al. 2018) and Betascan1* test (Siewert and Voight 2017) in 10 kb windows to each of the five populations. Genomic windows that were outliers for the Betscan* test, were enriched for low *NCD2* scores and high Tajima’s *D* values, indicating that these three metrics were largely complementary (supplementary fig. 12, Supplementary Material online).

### Are Targets of BS Shared Between Populations?

In some circumstances BS can be maintained as populations or species diverge (Siewert and Voight 2017; Bitarello et al. 2018; Mérot et al. 2020; Wang et al. 2020; Ding et al. 2021). Given these examples, we examined whether any genes had maintained BS between populations of the *L. donovani* species complex, indicating long-term BS. To assess this without relying on shared polymorphisms, we used the Betascan1* maximum and *NCD2* minimum scores for each gene, for each population as a summary statistic (see Materials and Methods). We find scant evidence for shared BS from Betascan1* scores. We define Betascan1* outlier genes as those in the upper 5% of Betascan1* scores for their population. There was little overlap in these outliers; 701 genes are outliers in at least one population, only 42 of these (6%) were outliers in two or more populations (supplementary table 3, Supplementary Material online), and only 9 are outliers in three or more populations (1%). The *NCD2* metric identified more overlap between populations, but long-term BS is still the exception; 1,627 genes were 5% outliers in at least one population and only 195 (11%) were outliers in more than one. However, because the *NCD2* metric measures the similarity of allele frequencies to a target frequency (0.5 in our case; Bitarello et al. 2018), genes that are merely subject to weaker purifying selection will have elevated *NCD2* scores.

Another possibility is that weak polygenic BS operates on a number of genes, perhaps transiently. This may be the case for frequency-dependent BS, for example, in exported and cell surface-located erythrocyte membranes and exported proteins in *Plasmodium falciparum* (Volkman et al. 2002; Jeffares et al. 2007; Claessens et al. 2014). In this scenario, we might expect BS targets in one population to predict genes with higher metrics in other populations, due to a history of weak BS. For example, if multiple genes are weakly influenced by BS, they may have elevated signals as a group, even if any one particular gene is not significant alone. We examined this using only Betascan1*, because we suspect that the measure of correlated allele frequency that Betascan utilizes will be less confounded by weak purifying selection. To examine this, we examined whether the 10 kb regions with the 5% highest Betascan1* scores in the East African EA1 population were enriched for high Betascan* scores (compared with the remainder of the genome). We found that outliers from EA1 do not show significantly elevated Betascan1* scores in any other population. We repeated this analysis for the 5% highest outliers from ISC1 and from BM (again comparing to each other population; supplementary fig. 18, Supplementary Material online). In almost all cases, outliers are not shared between populations. The only enrichment, that indicates common sites of BS between populations, was between the ISC populations, where ISC1 outliers predict higher scores in ISC2 (*P* = 2 × 10^−4^; supplementary fig. 18, Supplementary Material online). Since the ISC1 population has been derived from ISC2 population relatively recently (Imamura et al. 2016), we can expect some aspects of the genetic diversity to be maintained. In summary, signals of BS are generally not shared between populations of *L. donovani*.

### Identifying Genes That are Subject to BS

To advance research in *Leishmania* it would be useful to identify the most likely targets of BS. To achieve the most comprehensive detection of BS signatures, we performed both Betascan* and NCD2 tests with 1, 5, and 10 kb windows. We observed complete overlap between tests using 1 and 10 kb windows, with four additional ORF-containing regions identified with 5 kb windows (supplementary table 4 and fig. 16, Supplementary Material online). As a pragmatic approach, we sought to identify genes with robust and strong signatures from multiple metrics. To achieve this, we selected 10 kb genomic windows that were in the first or 99th percentile of either the *NCD2* test or *Betascan* tests, respectively. To identify the genes within *NCD2*/*Betascan* windows that are likely targets, we calculated nucleotide diversity (π) and Tajima’s *D* (Tajima 1989) for each gene, and selected genes in the 90th percentile of either statistic as well-supported plausible targets. We then selected genes that were outliers in both categories (NCD2 or Betascan* and π or Tajima’s *D*). This intersection identified 38 genes (supplementary table 4, Supplementary Material online). We manually vetted these to remove “hitchhikers,” genes whose high diversity was likely due to their proximity to a BS “driver” gene, resulting in 24 vetted candidates. We also removed genes with suspicious read coverage, because gene duplications produce strong artefactual signals of BS (supplementary fig. 13, Supplementary Material online). Due to the stringent process of filtering, this method is not guaranteed to have equal power to detect BS in the five populations we examine, which are represented by different numbers of isolates, have different levels of nucleotide diversity and different allele frequency distributions (fig. 2 and table 1).

**Fig. 3.**
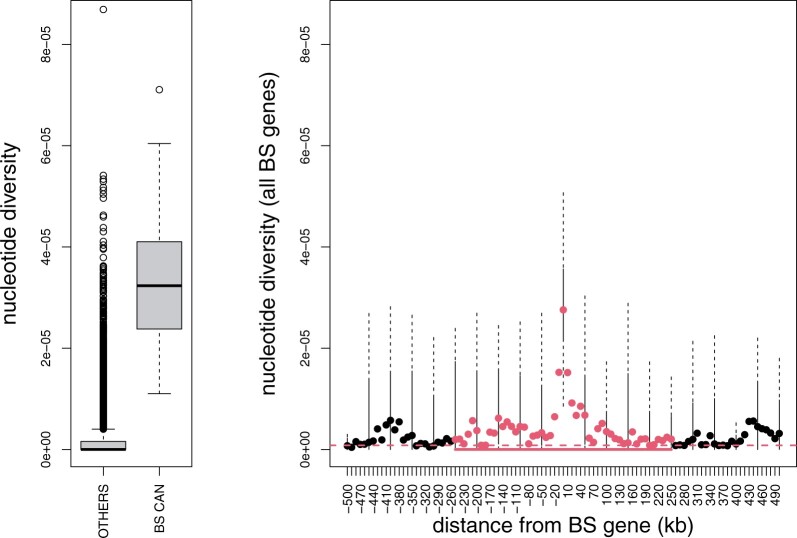
Diversity is significantly elevated in BS target regions. On the left we show the distribution of nucleotide diversity (π) genome-wide for the EA1 population (GW) and the distribution for the 500 kb around all the 20 vetted BS targets discovered in the EA1 population. On the right, the filled circles show the median π (for all BS targets) every 10 kb up and downstream to 500 kb from the targets. Circles are red where the diversity at this distance is significantly higher than the genome-wide distribution and black otherwise (Wilcoxon signed rank tests <1.5 × 10^−4^, using both up- and downstream π values). The distribution of nucleotide diversity values for target genes is shown using box and whisker plots at 50 kb intervals.

This screen identified 24 candidate genes (table 2; justification for vetting genes in supplementary table 4, Supplementary Material online). Candidate genes in the EA1 population, where the most were discovered, have nucleotide diversity that is 34-fold higher than the genome-wide median (fig. 3). Diversity is elevated in genome regions surrounding these target genes and remains significantly elevated up to 250 kb from the targets. Because the mean size for chromosomes in *L. donovani* is 900 kb, this increase in diversity influences a large proportion of the genome. Furthermore, BS candidates are enriched for high minor allele frequency (MAF) cosegregating sites and show higher levels of statistical linkage than genome-wide distributions, consistent with expectations for genes that are subject to BS (Charlesworth 2006; supplementary fig. 14, Supplementary Material online).

**Table 2:** Candidates for Genes Subject to Balancing Selection in the *Leishmania donovani* Complex

Candidate Gene	Description	Population	Tajima’s *D*	Variants (Nonsyn/Synon)	Tests NCD2/Beta
LdBPK_161760.1	FLAM3, flagellum attachment protein in *L. mexicana* (see Sunter et al. 2019)	ISC2	3.1	12/4	NCD2
LdBPK_341740.1	Zeta toxin protein 1, conserved in trypanosomes (see Srivastava et al. 2019)	EA2	3.3	35/18	Both
LdBPK_363870.1	Mitogen activated kinase-like protein, conserved in trypanosomes	EA1	3.9	10/9	Both
LdBPK_291600.1	Nodulin-like, conserved in trypanosomes	ISC2	3.2	8/9	Both
LdBPK_170210.1	Unknown function, conserved in *Leishmania*	EA1	3.0	6/9	Beta
LdBPK_261240.1	FYVE zinc finger containing protein, conserved in *Leishmania*	EA1	4.3	9/15	Both
LdBPK_262120.1	Putative kinase domain, conserved in *Leishmania*	EA1	3.9	7/23	Both
LdBPK_280190.1	Unknown function, conserved in *Leishmania*, contains helix domains	EA1	2.9	12/3	Beta
LdBPK_282030.1	p21-C-terminal region-binding protein, conserved in Trypanosomes	EA1	1.9	9/6	Both
LdBPK_301540.1	Rad17 cell cycle checkpoint clamp protein (hypothetical protein on TriTrypDB), conserved in trypanosomes, involved in chromatin binding, and DNA repair (see Nunes et al. 2011)	EA1	4.0	8/14	Both
LdBPK_302020.1	Unknown function, conserved in *Leishmania*	EA1	3.7	3/6	Both
LdBPK_311120.1	emp24/gp25L/p24/GOLD family, conserved in trypanosomes, involved in golgi vesicle transportation	EA1	2.9	4/2	Both
LdBPK_311710.1	Unknown function, conserved in *Leishmania*	EA1	3.8	8/9	Both
LdBPK_311170.1	Unknown function, conserved in *Leishmania*, adenylate cyclase regulatory protein-like	ISC2	3.1	8/2	Both
LdBPK_312260.1	Unknown function, conserved in *Leishmania*	EA1	4.3	20/6	Both
LdBPK_312550.1	2Fe–2S iron–sulfur cluster binding domain, only conserved in *L. donovani* and *L. infantum*, cofactor, implicated in redox metabolism (see Kumar et al. 2019)	EA1	4.3	15/4	Beta
LdBPK_330840.1	Nuclear LIM interactor-interacting (NLI) factor-like phosphatase, conserved in *Leishmania*	EA1	4.8	27/17	Both
LdBPK_350960.1	Unknown function, conserved in trypanosomes	EA1	2.6	3/4	Both
LdBPK_361900.1	Ras-like small GTPase, conserved in *Leishmania*	EA1	3.7	6/3	NCD2
LdBPK_363830.1	Unknown function—shares >40% similarity with tectonic/cilia protein, conserved across trypanosomes (see Dean et al. 2016)	EA1	3.8	6/8	Both
LdBPK_365550.1	Glutathione S-transferase domain containing protein, conserved in trypanosomes	EA1	3.8	7/3	Both
LdBPK_366210.1	Unknown function, conserved in *Leishmania*	EA1	4.1	6/8	Both
LdBPK_300960.1	Hypothetical protein, conserved in *Leishmania*	EA1	4.2	10/10	Beta
LdBPK_312990.1	Clathrin and VPS/zinc finger RING-type	EA1	4.3	14/11	Both

Consistent with the lack of evidence for shared BS between populations, the genes that are BS candidates in the EA1 population do not show significantly elevated Tajma’s *D* values in any other population (supplementary fig. 17, Supplementary Material online).

To identify these candidates NCD2 and Betscan* tests were applied in 10 kb genomic windows for each population. Candidate genes that were both 1) outliers for at least one test (>99.5 percentile) and 2) outliers (95 percentile) for either Tajima’s D and/or nucleotide diversity (π, calculated within the gene start-end window). Details of the method are described in supplementary fig. 6 and text 2, Supplementary Material online. All comparisons of tests for each population are available in supplementary figures 7–11, Supplementary Material online, respectively. Where protein function is “unknown” on TriTrypDB, we subjected each protein to BLASTp searches to obtain homology to other known proteins and ascertain conservation across trypanosomes.

### Candidate Genes for BS in the *L. donovani* Complex

**Fig. 4.**
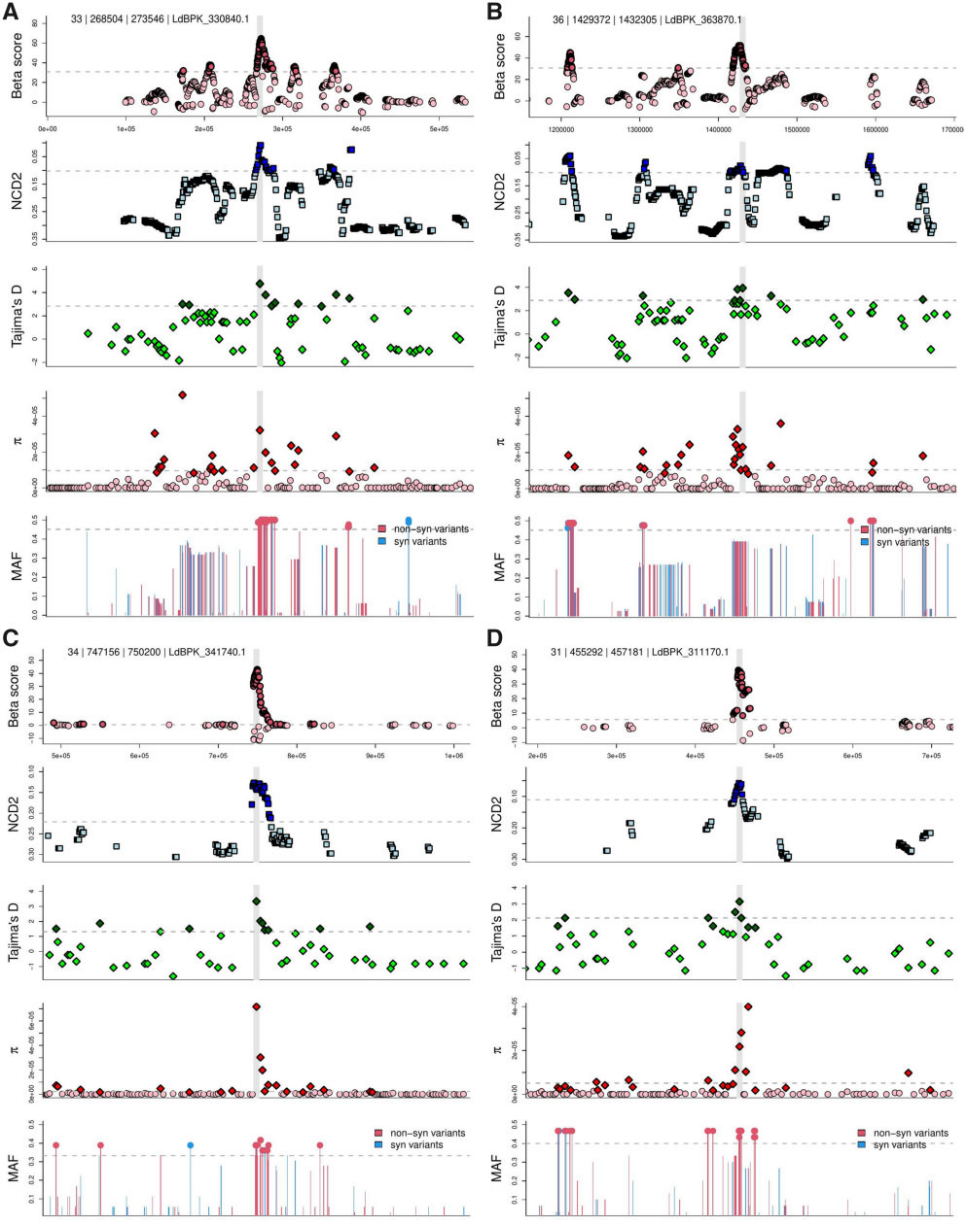
Candidate genes show multiple genetic signatures of balancing selection. We show Betascan*, *NCD2*, Tajima’s *D*, nucleotide diversity (π), and minor allele frequency (MAF) in a 250 kb window around four candidate genes. The location of the candidate gene is indicated by a vertical gray bar. The population-specific 90th percentile for each metric is shown as a horizontal dashed line, scores that are above this are drawn in darker shades, or plotted with a filled dot for MAF. Panel titles indicate the chromosome, gene start and end coordinates, and gene ID. Genes and populations where BS detected are; (*A*) NLI interacting factor-like phosphatase LdBPK_330840.1 (EA1); (*B*) mitogen activated kinase-like protein LdBPK_363870.1 (EA1); (*C*) putative Zeta toxin LdBPK_341740.1 (EA2); (*D*) hypothetical protein LdBPK_311170.1 (ISC2). Similar plots are shown for all candidate genes in supplementary figure 16, Supplementary Material online.

Our manual vetting of BS candidates retained 24 genes (table 2), of which 20 were discovered in the EA1 population. We did not discover any reliable candidates in the two populations that appear to be expanding following a bottleneck (BM and ISC1). Figure 4 illustrates the variety of robust genetic signatures that implicate four of these genes. All vetted genes contain similarly robust signatures (supplementary fig. 16, Supplementary Material online).

Several genes caught our attention as interesting targets of selection. LdBPK_291600.1 encodes a transmembrane protein containing a nodulin-like domain. Such proteins have been implicated in membrane transport and iron homeostasis (Laranjeira-Silva et al. 2018) in *Leishmania*. The zeta toxin domain protein (LdBPK_341740.1; fig. 4) is indicated as a BS target by both Betascan* and *NCD2* metrics in the East African population EA2. The gene also has high nucleotide diversity in EA1 (supplementary table 5, Supplementary Material online). Because the phylogeny of this zeta toxin gene does not separate EA1 and EA2 isolates, as we would expect from the genome-scale divergence in figure 1, this gene may be subject to BS in both species, or may be a recent instance of between-population introgression. The zeta domain is positioned at 744–861aa, with two nonsynonymous variants resulting in the changes Leu747Thr and Ser752Phe, respectively, from the reference genome. The zeta toxin is part of the Type-II toxin–antitoxin (TA) module identified in prokaryotes, with homologues only recently discovered in *Leishmania* (Srivastava et al. 2019). The toxin component of the TA module acts against cellular processes such as translation and is neutralized by the antitoxin component in favorable conditions. Sharing similar functional domains and activity with the *E. coli* homologue (Srivastava et al. 2019), *L. donovani* zeta toxin may therefore also be implicated in stress response and/or virulence (Rocker and Meinhart 2015).

The *FLAM3* gene (LdBPK_161760.1) encodes a flagellum attachment zone protein essential for host interaction (Sunter et al. 2015, 2019; An and Li 2018). Our strict criteria have indicated this gene as under BS only in the ISC population ISC2, but it also have high nucleotide diversity in four of the five populations (supplementary table 5, Supplementary Material online). The *FLAM3* protein contains a clustered mitochondria (CLU) domain and a domain of repeats (Sunter et al. 2015). The majority of variants in ISC2 occur between these domains, with none falling within the CLU domain (supplementary table 6, Supplementary Material online).

## Discussion

Here, we sequenced 93 strains of *L. infantum* from Brazil, contributing to a worldwide collection of *L. donovani* complex isolates along with previous analyses (Imamura et al. 2016; Carnielli et al. 2018; Zackay et al. 2018; Franssen et al. 2020). Our analysis of this population is consistent with these previous studies, showing that the *L. infantum* population in Brazil contains very little genetic diversity (Carvalho et al. 2020; Schwabl et al. 2021). A consistent observation in analysis of this species complex is that populations from East Africa, India, and Brazil are substantially genetically differentiated, a result that we reiterate here (fig. 1). In this study of BS, we also show that signals of selection largely differ between populations.

Relatively few studies have attempted BS screenings in parasites (reviewed by Weedall and Conway 2010). Our study uses the Betascan* (Siewert and Voight 2017) and *NCD2* (Bitarello et al. 2018) metrics that have been developed recently and have been shown to outperform classic metrics under models of BS, such as Tajima’s *D* (Tajima 1989). These analytic tools, and the use of multiple populations should produce an analysis at least as sensitive as previous screens for BS searches within parasites such as *Plasmodium*, producing up to 25 candidate genes (Tetteh et al. (2009), 6 genes; Amambua-Ngwa et al. [2012], 25 genes; Nygaard et al. [2010], 19 genes).

The 24 candidate genes uncovered here possess varying functions within the *L.donovani* species complex. The flagellum attachment gene *FLAM3* is a striking candidate considering the importance of the protein in parasite cellular structure, proliferation, and differentiation (Hayes et al. 2014; Sunter et al. 2019; Halliday et al. 2020). Furthermore, candidate genes LdBPK_311120.1 and LdBPK_361900.1, which encode a member of the emp24/gp25L/p24/GOLD family and a ras-like GTPase, respectively, may influence trafficking of virulence factors, and subsequently interaction with their host. ras-like GTPases may contribute to attenuation of VL via the TOR pathway in *L. donovani* (Zhang et al. 2014). Given the lack of detailed studies of the majority of these candidates in the *L. donovani* species complex, studies of the cell biology of these proteins will be useful next steps.

Our analysis suggests that the performance of Betascan* and *NCD2* BS tests are dependent on the changes of population demography within this species complex. This is partly due to our pragmatic screening criteria that required >5 variants per genomic window (Betascan*, *NCD2*) and >5 variants per gene (Tajima’s *D* and π metrics). The low-diversity Brazil and ISC1 populations contain far fewer regions that satisfy these criteria. However, strong population bottlenecks would enhance the loss of polymorphic sites by drift, ablating strong BS signatures. For example, 99% of 1 kb genomic windows in our BM population contain <5 segregating sites, which will reduce the scale of Betascan* and *NCD2* metrics. This could result in a loss of power to detect long-term BS. Only two populations (EA1 and ISC2) appear to have relatively stable population sizes and sufficient nucleotide diversity to identify BS candidates using our pragmatic methods (table 1). More complex methods that employ population models to detect BS are available (DeGiorgio et al. 2014; Cheng and DeGiorgio 2019, 2020). We chose not to employ these because we do not believe that *L. donovani* complex populations are sufficiently understood to be meaningfully modeled at this stage. For example, there is no accurate estimate of the recombination rates or mutation rates in these populations, nor estimates of divergence between *L. donovani* and *L. infantum* in terms of generations since it is not clear how many generations per year *Leishmania* species undergo in natural conditions. Our approach was to divide the samples into multiple populations and exclude potential between-population hybrids, with the expectation that this would alleviate some of the issues resulting from population structure.

We conclude that BS targets are generally not conserved between populations of the *L. donovani* complex. rather than a lack of power. The differentiation of BS targets is most likely due to differentiated processes (or history) of selection, rather than a lack of power to detect targets in some populations. Several observations led us to this view. Tajima’s *D* is not at all correlated between populations (data not shown), but this might be expected if most genes are dominated by drift. The high *F*_ST_ between populations, and relatively few shared polymorphisms are consistent with this. The initial 24 candidate BS genes that were discovered in the EA1 population, did not have statistically higher Tajima’s *D* (as a group) in any other population (supplementary fig. 17, Supplementary Material online), so this set is not enriched for BS elsewhere. We also examined whether Betascan* outlier genes in EA1, ISC1, or BM were enriched in any other population (supplementary fig. 18, Supplementary Material online). In general, outliers in one population were not enriched for Betascan* scores in any other population, the only exception was ISC1 outliers were enriched in ISC2. Given the relatively recent derivation of ISC1 from ISC2 in the 1970s (Imamura et al. 2016), some maintenance of diversity is to be expected.

It is possible that other evolutionary changes caused some of the signals we observe, including introgression events, partial selective sweeps or transient heterozygosity excess that can occur as a consequence of adaptation (Sellis et al. 2011). Local adaptation can also lead to an appearance of within-population diversity and/or excess heterozygosity (Wood et al. 2008; Eizaguirre and Lenz 2010; Ellison et al. 2011; Keller et al. 2011), particularly when the distribution of local “niches” is not well understood, which is generally the case with *Leishmania*. It is possible, for example, that multiple variants exist within these genes as adaptations to regional differences in host or sandfly cellular/extracellular environments. Parasite genes may vary with regional variations in HLA loci that affect susceptibility to VL (Blackwell et al. 2020). In any case, the candidate genes we identify warrant further study.

It is possible that BS of single-copy genes is not the most important mechanism that maintains diversity within the *L. donovani* complex, or protozoan parasites generally. The effects of multicopy gene families encoding RIFIN, STEVOR, and PfEMP1 variant surface antigens in pathogenesis of *Plasmodium* parasites are well-described (e.g., Claessens et al. 2014; Wahlgren et al. 2017). These genes are typically removed from BS screens, because multicopy genes will produce artefactual signals of BS when analyzed with current bioinformatics methods (supplementary fig. 15, Supplementary Material online). Although variants in duplicated regions or multiple copies of genes may allow the parasite to maintain diversity, it is an open question whether this diversity is maintained by neutral processes or BS.

At present we regard our candidate genes as “likely suspects” for BS, rather than experimentally proved examples. There are various biological scenarios that could produce signals of BS. Perhaps the simplest is frequency-dependent rare allele advantage or overdominance within human/mammalian hosts. In this case, experimental support for these targets would require demonstration that host cell populations produced different responses to different alleles of the proteins. Another possibility is that overdominance is caused by alleles whose fitness differs in sandfly and human hosts. Technically, this is more challenging to test, but could be achieved by tracking genotype frequencies of segregating F_2_ populations within laboratory passages between sandfly and mammalian models. Finally, signals of BS can be caused by fine-scale clines of alleles with differential fitness across different environments (Westram et al. 2021). In our case, these could be sand fly or human host genotypes. Evidence for this scenario would require fine-scale localized genetic data.

In summary, our description of diversity in the *L. donovani* species complex provides insight into the global populations of this parasite. We show that these populations are genetically divergent, with independent signals of BS. Our discovery of a handful of genes with robust signatures of BS provides candidate genes for the study of host–parasite and host–vector interactions.

## Materials and Methods

### Ethics

Samples from Brazil were obtained as part of a broad study for genomic studies in the Laboratory of Leishmaniasis at the Institute of Tropical Medicine Natan Portella, approved by the Research Ethics Committee of the Federal University of Piauí (approval ID number 0116/2005). All methods were performed according to the approved guidelines and regulations. A written informed consent was obtained from all study participants or their legal guardians.

### Strain Culture and Genome Sequencing

Bone marrow aspirates were obtained from the routine diagnosis of patients admitted to the Natan Portella Tropical Diseases Institute in Teresina-PI, Brazil. Aspirates were inoculated into a mixed culture medium NNN (Neal, Novy, Nicolle) containing 2 ml of Schneider’s medium supplemented with 10% fetal bovine serum, 2% urine and penicillin 10,000 U/ml, and streptomycin 10 mg/ml. The positive isolates in mixed media were expanded in Schneider’s liquid medium under the same conditions mentioned above. Extraction of DNA from the parasites was performed after washing to remove culture medium, using Qiagen Blood and Tissue kit was used according to the manufacturer’s recommendation.

Genome sequencing was performed on Illumina HiSeq 2500 machines (or similar) to produce paired end 150 nt reads. The majority (95%) of the samples were sequenced to provide mapped read coverage of ≥30× (mean 97×, minimum 19×). Raw sequencing reads were submitted to NCBI’s sequencing read archive under the BioProject accession PRJNA702997.

### Sequence Analysis/Variant Calling

Publicly available *L. donovani* complex data were downloaded in FASTQ format from the European Nucleotide Archive (ENA: https://www.ebi.ac.uk/ena). Full list of strain names/ENA numbers in supplementary table 1, Supplementary Material online. The *L. donovani* reference genome (strain BPK282A1) was downloaded from TriTrypDB (version November 2019). Strain reads were mapped to the reference using bwa v.0.7.17 (Li and Durbin 2009), converted to bam, sorted, indexed, and duplicates removed with SAMtools v.1.9 (Li et al. 2009).

For each strain, SNPs and indels were called using The Genome Analysis Toolkit (GATK) HaplotypeCaller v.4.1.0.0 (Depristo et al. 2011) using the “discovery” genotyping mode Freebayes v.1.3.2 (https://github.com/ekg/freebayes) accepting calls with a minimum alternative allele read count ≥5. We accepted calls discovered by both methods, merged all VCFs and regenotyped with Freebayes. The regenotyped VCF was sorted with Picard SortVcf (https://broadinstitute.github.io/picard/) and indexed with GATK IndexFeatureFile. SNP hard-filtering was performed with BCFtools (https://samtools.github.io/bcftools/) on biallelic variants only, to remove sites with any of the following: DPRA < 0.73 or > 1.48; QA or QR < 100; SRP or SAP > 2,000; RPP or RPPR > 3,484; PAIRED or PAIRPAIREDR < 0.8; MQM or MQMR < 40. As chromosome 31 is generally supernumerary, we specified DPB < 3,0401 or > 121,603 to be removed, and for remaining chromosomes, DPB < 182,99 or > 73,197 (<0.5× or >2× median DPB). Biallelic indels were filtered to remove sites with any of the following: DPRA < 0.73 or > 1.48; QA or QR < 100; SRP or SAP > 2,000. VCF annotation was performed with the snpEff v.4.3 package (Cingolani et al. 2012) using the default Leishmania_donovani_BPK282A1 database included with the software. SnpSift filter with the option “ANN[*].EFFECT has ‘missense_variant’” was used to extract nonsynonymous sites.

With this variant filtering we observed a correlation between MAF and read coverage at SNP sites (supplementary fig. 13, Supplementary Material online). Modeling showed that duplications resulted in a systematic bias against calling rare alleles. We therefore removed any SNP/indel sites where the mean variant coverage within the ADMIXTURE-defined population was ≥1.5× larger than the median coverage (corresponding to triploid sites in a generally diploid chromosome), or ≥1.25× larger than the median coverage for chromosome 31 (corresponding to tetraploid sites in a generally triploid chromosome). We also removed sites where coverage was highly variable, by excluding sites in the upper 5th percentile of the coverage standard deviation (SD). In each population this filtered ∼5–7% of sites. Mapping coverage was ascertained by SAMtools bedcov for each gene in the multipopulation Variant Call Format (VCF) file. After this filtering, the correlation between MAF and read coverage was either far less significant or removed completely. This filtering retained 10,377 out of a possible 10,778 sites in population ISC1; 9,781 out of a possible 10,227 sites in population ISC2; 40,127 out of a possible 41,957 sites in EA1; 11,757 out of a possible 12,365 sites in EA2, and 26,884 out of a possible 28,281 sites in BM.

To validate the variant filtering we produced a de novo assembly of the MHOM/BR/06/MA01A *L. infantum* isolate from Brazil (Carnielli et al. 2018), mapped Illumina reads from the same isolate to the assembly, and called SNPs and indels as above. All calls should be heterozygous sites, or errors. Initial variant calling identified 4 SNPs and 23 indels, after filtering no SNPs or indels remained, consistent with a very low false positive call per strain. The MHOM/BR/06/MA01A de novo assembly will be described elsewhere. Briefly, the assembly was produced using Oxford Nanopore Technology (ONT) reads to 110× coverage, assembled with Canu v.1.9 (Koren et al. 2017), polished once using ONT reads using Nanopolish v.0.9.2 (Loman et al. 2015), and thrice with Illumina reads using Pilon v.1.22 (Walker et al. 2014).

### Phylogenetic Analysis

VCF containing all variants from all 477 isolates was converted to PHYLIP format using vcf2phylip (available at https://github.com/edgardomortiz/vcf2phylip/tree/v2.0). This produced an alignment of 283,378 sites. IQ-TREE v.1.5.5 (Nguyen et al. 2015) was used to perform maximum likelihood (ML) phylogenetic analysis with the model GTR + ASC, which includes ascertainment bias correction, with 1,000 bootstrap replicates and 1,000 UFBOOT (Hoang et al. 2018) approximations to produce ML support values. The resulting tree was visualized with Figtree v.1.4.4 (available at http://tree.bio.ed.ac.uk/). Treefiles are available in supplementary material posted to FigShare online.

### Population and Diversity Analysis

For all population analyses we utilized only biallelic SNPs, pruning linked sites (*r*^2^ > 0.5) in 2 kb windows with a step size of 1 with PLINK v.1.9 (Purcell et al. 2007) using the option –indep-pairwise 2 kb 1 0.5. This produced 194,351 SNPs from the initial 353,301 (158,950 variants removed). ADMIXTURE v.1.3 (Alexander and Lange 2011) was run, unsupervised, with *K* = 1–12. Principal component coordinates were produced with PLINK v.1.9.

Prior to BS tests performed on the five populations (EA1, EA2, ISC1, ISC2, BM), mixed ancestry strains were removed from population VCFs. Population-specific VCFs were filtered with VCFtools v.0.1.15 (Danecek et al. 2011) to remove sites that were fixed within a population (option –mac 1). Repeat regions (see below) were also filtered out of VCFs at this stage. Tajima’s *D*, π, and MAF were calculated on unpruned variants using VCFtools. Tests for BS used biallelic SNPs and indels from each population. Copy-number variant and duplicated genome regions were removed from this analysis, as these regions will produce biases in allele frequencies toward common alleles, producing artifactual signals of BS (supplementary text 1 and fig. 5, Supplementary Material online). Variant calling for multicopy regions was beyond the scope of this study.

Repeat regions were determined as follows. Intergenic coordinates in *L. donovani* were extracted from the annotation .gff, downloaded from TriTrypDB (version November 2019) with BEDtools v.2.27.1 (Quinlan and Hall 2010) complement with default parameters. Intergenic regions were then extracted from the genome using BEDtools getfasta. Repeat regions in *L. donovani* were identified by nBLASTing v.2.7.1 (Altschul et al. 1990) intergenic regions against the rest of the genome, disregarding redundant hits and those <200nt in length. Resulting coordinates were converted to bed format for filtering out of the VCF. This filtering removed 401 sites in the ISC1 population; 446 in ISC2; 1,830 in EA1; 608 in EA2, and 1,387 in BM.

### BS Tests

The NCD2 test used Equation 1 provided by Bitarello et al. (2018), using windows of 1, 5, and 10 kb with step sizes of 0.5, 2.5, and 5 kb, respectively. Ten kilobase pairs of window sizes were used in this study as sizes of 1 and 5 kb largely returned windows without scores (but see Identifying Genes that are Subject to BS). A list of fixed differences between *L. donovani* populations (total 285 isolates) and the nonadmixed BM *L. infantum* population (127 isolates) was used in the analysis for NCD2: Fixed difference sites were encoded as MAF = 0. Fixed differences were determined using bcftools isec called on VCFs of all *L. donovani* populations and *L. infantum* containing fixed variants, resulting in 76,284 fixed difference sites. Our target frequency of 0.5 (*tf*) and Equation 2 of Bitarello et al. (2018) were used to generate *Ztf-IS* scores, with the exception of using the SD for each number of informative sites (IS) rather than simulated SD. *P* values for each window were calculated by assigning a rank based on *Ztf-IS* score and dividing by the total number of scanned windows.

The Betascan1* test (Siewert and Voight 2017), using default parameters, using the file format generated from the variants using glactools (Renaud 2018; available at: https://github.com/grenaud/glactools) We performed the test on each individual population.

Betascan1* and *NCD2* scores were calculated in windows around each variant site. To obtain values for each gene, we used the maximum Betascan1* score for all variants within the gene and the minimum *NCD2* score within each gene (because low *NCD2* scores are indicators of BS). Following the recommendations of Siewert and Voight (2020), we only considered windows containing ≥5 variants.

### Gene Ontology Analysis

Gene Ontology (GO) descriptions and gene details for the *L. donovani* BPK282A1 reference genome were downloaded from TriTrypDB. GO enrichment analysis was performed using the PANTHER service on tritrypdb.org. Proteins that were classed as “hypothetical” or of “unknown function” were BLASTed against the nonredundant protein sequences (nr) database of NCBI to obtain possible identity by shared homology, and to determine conservation across trypanosomes.

## Supplementary Material

Supplementary data are available at *Genome Biology and Evolution* online.

## Supplementary Material

evab265_Supplementary_Data

## References

[evab265-B1] Alexander DH , Lange K. 2011. Enhancements to the ADMIXTURE algorithm for individual ancestry estimation. BMC Bioinformatics 12:246. 21682921 10.1186/1471-2105-12-246 PMC3146885

[evab265-B2] Altschul SF , Gish W, Miller W, Myers EW, Lipman DJ. 1990. Basic local alignment search tool. J Mol Biol. 215(3):403–410. 2231712 10.1016/S0022-2836(05)80360-2

[evab265-B3] Amambua-Ngwa A , et al 2012. Population genomic scan for candidate signatures of balancing selection to guide antigen characterization in malaria parasites. PLoS Genet. 8(11):e1002992. 23133397 10.1371/journal.pgen.1002992 PMC3486833

[evab265-B4] An T , Li Z. 2018. An orphan kinesin controls trypanosome morphology transitions by targeting FLAM3 to the Flagellum. PLoS Pathog. 14(5):e1007101. 29813136 10.1371/journal.ppat.1007101 PMC5993322

[evab265-B5] Atayde VD , et al 2016. *Leishmania* exosomes and other virulence factors: impact on innate immune response and macrophage functions. Cell Immunol. 309:7–18. 27499212 10.1016/j.cellimm.2016.07.013

[evab265-B6] Bhattacharya SK , Dipika Sur PKS, Karbwang J. 2006. Elimination of Leishmaniasis (kala-Azar) from the Indian subcontinent is technically feasible & operationally achievable. Indian J Med Res. 123(3):195–196. 16778303

[evab265-B7] Bitarello BD , et al 2018. Signatures of long-term balancing selection in human genomes. Genome Biol Evol. 10(3):939–955. 29608730 10.1093/gbe/evy054 PMC5952967

[evab265-B8] Blackwell JM , Fakiola M, Castellucci LC. 2020. Human genetics of *Leishmania* infections. Hum Genet. 139(6–7):813–819. 32055998 10.1007/s00439-020-02130-w PMC7272388

[evab265-B9] Boité MC , et al 2019. Trans-Atlantic spill over: deconstructing the ecological adaptation of *Leishmania infantum* in the Americas. Genes 11(1):4. 10.3390/genes11010004 PMC7017240 31861501

[evab265-B10] Carnielli JBT , et al 2018. A *Leishmania infantum* genetic marker associated with Miltefosine treatment failure for visceral Leishmaniasis. EBioMedicine 36:83–91. 30268832 10.1016/j.ebiom.2018.09.029 PMC6197651

[evab265-B11] Carvalho KSS , et al 2020. Application of next generation sequencing (NGS) for descriptive analysis of 30 genomes of *Leishmania infantum* isolates in Middle-North Brazil. Sci Rep. 10(1):12321. 32704096 10.1038/s41598-020-68953-9 PMC7378178

[evab265-B12] Charlesworth D. 2006. Balancing selection and its effects on sequences in nearby genome regions. PLoS Genet. 2(4):e64. 16683038 10.1371/journal.pgen.0020064 PMC1449905

[evab265-B13] Cheng X , DeGiorgio M. 2019. Detection of shared balancing selection in the absence of trans-species polymorphism. Mol Biol Evol. 36(1):177–199. 30380122 10.1093/molbev/msy202 PMC6530816

[evab265-B14] Cheng X , DeGiorgio M. 2020. Flexible mixture model approaches that accommodate footprint size variability for robust detection of balancing selection. Mol Biol Evol. 37(11):3267–3291. 32462188 10.1093/molbev/msaa134 PMC7820363

[evab265-B15] Cingolani P , et al 2012. A program for annotating and predicting the effects of single nucleotide polymorphisms, SnpEff: SNPs in the genome of *Drosophila melanogaster* strain w1118; Iso-2; Iso-3. Fly (Austin) 6(2):80–92. 22728672 10.4161/fly.19695 PMC3679285

[evab265-B16] Claessens A , et al 2014. Generation of antigenic diversity in *Plasmodium falciparum* by structured rearrangement of var genes during mitosis. PLoS Genet. 10(12):e1004812. 25521112 10.1371/journal.pgen.1004812 PMC4270465

[evab265-B17] Cotton JA , et al 2020. Genomic analysis of natural intra-specific hybrids among Ethiopian isolates of *Leishmania donovani*. PLoS Negl Trop Dis. 14(4):e0007143. 32310945 10.1371/journal.pntd.0007143 PMC7237039

[evab265-B18] Danecek P , et al; 1000 Genomes Project Analysis Group. 2011. The variant call format and VCFtools. Bioinformatics 27(15):2156–2158. 21653522 10.1093/bioinformatics/btr330 PMC3137218

[evab265-B19] Dean S , Moreira-Leite F, Varga V, Gull K. 2016. Cilium transition zone proteome reveals compartmentalization and differential dynamics of ciliopathy complexes. Proc Natl Acad Sci U S A. 113(35):E5135–E5143. 27519801 10.1073/pnas.1604258113 PMC5024643

[evab265-B20] DeGiorgio M , Lohmueller KE, Nielsen R. 2014. A model-based approach for identifying signatures of ancient balancing selection in genetic data. PLoS Genet. 10(8):e1004561. 25144706 10.1371/journal.pgen.1004561 PMC4140648

[evab265-B21] Depristo MA , et al 2011. A framework for variation discovery and genotyping using next-generation DNA sequencing data. Nat Genet. 43(5):491–498. 21478889 10.1038/ng.806 PMC3083463

[evab265-B22] Dhiman RC , Yadav RS. 2016. Insecticide resistance in phlebotomine sandflies in Southeast Asia with emphasis on the Indian subcontinent. Infect Dis Poverty 5(1):106. 27817749 10.1186/s40249-016-0200-3 PMC5098277

[evab265-B23] Ding G , et al 2021. Global allele polymorphism indicates a high rate of allele genesis at a locus under balancing selection. Heredity (Edinb) 126(1):163–177. 32855546 10.1038/s41437-020-00358-w PMC7853069

[evab265-B24] Dong G , Filho AL, Olivier M. 2019. Modulation of host-pathogen communication by extracellular vesicles (EVs) of the protozoan parasite *Leishmania*. Front Cell Infect Microbiol. 9:100. 31032233 10.3389/fcimb.2019.00100 PMC6470181

[evab265-B25] Dumetz F , et al 2017. Modulation of aneuploidy in *Leishmania donovani* during adaptation to different in vitro and in vivo environments and its impact on gene expression. mBio 8(3):e00599-17. doi:10.1128/mBio.00599-17. PMC5442457 28536289

[evab265-B26] Dye C , Wolpert DM. 1988. Earthquakes, influenza and cycles of Indian Kala-Azar. Trans R Soc Trop Med Hyg. 82(6):843–850. 3256984 10.1016/0035-9203(88)90013-2

[evab265-B27] Eizaguirre C , Lenz TL. 2010. Major histocompatibility complex polymorphism: dynamics and consequences of parasite-mediated local adaptation in fishes. J Fish Biol. 77(9):2023–2047. 21133915 10.1111/j.1095-8649.2010.02819.x

[evab265-B28] Ellison CE , et al 2011. Population genomics and local adaptation in wild isolates of a model microbial eukaryote. Proc Natl Acad Sci U S A. 108 (7):2831–2836. 21282627 10.1073/pnas.1014971108 PMC3041088

[evab265-B29] Ferreira GEM , et al 2012. The genetic structure of *Leishmania infantum* populations in Brazil and its possible association with the transmission cycle of visceral Leishmaniasis. PLoS One 7(5):e36242. 22606248 10.1371/journal.pone.0036242 PMC3350531

[evab265-B30] Franssen SU , et al 2020. Global genome diversity of the *Leishmania donovani* complex. eLife 9:e51243. doi:10.7554/eLife.51243. PMC7105377 32209228

[evab265-B31] Gelanew T , et al 2014. Multilocus sequence and microsatellite identification of intra-specific hybrids and ancestor-like donors among natural Ethiopian isolates of *Leishmania donovani*. Int J Parasitol. 44(10):751–757. 24995620 10.1016/j.ijpara.2014.05.008 PMC4147965

[evab265-B32] Gelanew T , et al 2010. Inference of population structure of *Leishmania donovani* strains isolated from different Ethiopian visceral Leishmaniasis endemic areas. PLoS Negl Trop Dis. 4(11):e889. 21103373 10.1371/journal.pntd.0000889 PMC2982834

[evab265-B1530466] Guy AJ , et al 2018. Proteome-wide mapping of immune features onto Plasmodium protein three-dimensional structures. Sci Rep. 8(1):4355. 29531293 10.1038/s41598-018-22592-3 PMC5847524

[evab265-B33] Halliday C , et al 2020. Role for the flagellum attachment zone in *Leishmania* anterior cell tip morphogenesis. PLoS Pathog. 16(10):e1008494. 33091070 10.1371/journal.ppat.1008494 PMC7608989

[evab265-B34] Hayes P , et al 2014. Modulation of a cytoskeletal Calpain-like protein induces major transitions in trypanosome morphology. J Cell Biol. 206(3):377–384. 25092656 10.1083/jcb.201312067 PMC4121973

[evab265-B35] Hoang DT , Chernomor O, von Haeseler A, Minh BQ, Vinh LS. 2018. UFBoot2: improving the ultrafast bootstrap approximation. Mol Biol Evol. 35(2):518–522. 29077904 10.1093/molbev/msx281 PMC5850222

[evab265-B36] Hocking SE , Divis PCS, Kadir KA, Singh B, Conway DJ. 2020. Population genomic structure and recent evolution of *Plasmodium knowlesi*, Peninsular Malaysia. Emerg Infect Dis. 26(8):1749–1758. 32687018 10.3201/eid2608.190864 PMC7392424

[evab265-B37] Imamura H , et al 2016. Evolutionary genomics of epidemic visceral Leishmaniasis in the Indian subcontinent. eLife 5:56. 10.7554/eLife.12613 PMC4811772 27003289

[evab265-B38] Jeffares DC , et al 2007. Genome variation and evolution of the malaria parasite *Plasmodium falciparum*. Nat Genet. 39(1):120–125. 17159978 10.1038/ng1931 PMC2663918

[evab265-B39] Jeffares DC , et al 2015. The genomic and phenotypic diversity of *Schizosaccharomyces pombe*. Nat Genet. 47(3):235–241. 25665008 10.1038/ng.3215 PMC4645456

[evab265-B40] Keller SR , Levsen N, Ingvarsson PK, Olson MS, Tiffin P. 2011. Local selection across a latitudinal gradient shapes nucleotide diversity in *Balsam poplar*, *Populus balsamifera* L. Genetics 188(4):941–952. 21624997 10.1534/genetics.111.128041 PMC3176098

[evab265-B41] Koren S , et al 2017. Canu: scalable and accurate long-read assembly via adaptive K-Mer weighting and repeat separation. Genome Res. 27(5):722–736. 28298431 10.1101/gr.215087.116 PMC5411767

[evab265-B42] Kuhls K , et al 2011. Comparative microsatellite typing of new world *Leishmania infantum* reveals low heterogeneity among populations and its recent old world origin. PLoS Negl Trop Dis. 5(6):e1155. 21666787 10.1371/journal.pntd.0001155 PMC3110170

[evab265-B43] Kumar A , Chauhan N, Singh S. 2019. Understanding the cross-talk of redox metabolism and Fe-S cluster biogenesis in *Leishmania* through systems biology approach. Front Cell Infect Microbiol. 9:15. 30778378 10.3389/fcimb.2019.00015 PMC6369582

[evab265-B44] Laranjeira-Silva MF , et al 2018. A MFS-like plasma membrane transporter required for *Leishmania* virulence protects the parasites from iron toxicity. PLoS Pathog. 14(6):e1007140. 29906288 10.1371/journal.ppat.1007140 PMC6021107

[evab265-B45] Li H , Durbin R. 2009. Fast and accurate short read alignment with Burrows–Wheeler transform. Bioinformatics 25(14):1754–1760. 19451168 10.1093/bioinformatics/btp324 PMC2705234

[evab265-B46] Li H , et al; 1000 Genome Project Data Processing Subgroup. 2009. The sequence alignment/map (SAM) format and SAMtools. Bioinformatics 25(16):2078–2079. 19505943 10.1093/bioinformatics/btp352 PMC2723002

[evab265-B47] Loman NJ , Quick J, Simpson JT. 2015. A complete bacterial genome assembled de novo using only nanopore sequencing data. Nat Methods 12(8):733–735. 26076426 10.1038/nmeth.3444

[evab265-B48] Mérot C , Llaurens V, Normandeau E, Bernatchez L, Wellenreuther M. 2020. Balancing selection via life-history trade-offs maintains an inversion polymorphism in a seaweed fly. Nat Commun. 11(1):670. 32015341 10.1038/s41467-020-14479-7 PMC6997199

[evab265-B49] Mobegi VA , et al 2014. Genome-wide analysis of selection on the malaria parasite *Plasmodium falciparum* in West African populations of differing infection endemicity. Mol Biol Evol. 31(6):1490–1499. 24644299 10.1093/molbev/msu106 PMC4032133

[evab265-B50] Mullaney JM , Mills RE, Stephen Pittard W, Devine SE. 2010. Small insertions and deletions (INDELs) in human genomes. Hum Mol Genet. 19(R2):R131–R136. 20858594 10.1093/hmg/ddq400 PMC2953750

[evab265-B51] Muniaraj M. 2014. The lost hope of elimination of Kala-Azar (visceral Leishmaniasis) by 2010 and cyclic occurrence of its outbreak in India, blame falls on vector control practices or co-infection with human immunodeficiency virus or therapeutic modalities? Trop Parasitol. 4(1):10–19. 24754021 10.4103/2229-5070.129143 PMC3992795

[evab265-B52] Nguyen L-T , Schmidt HA, von Haeseler A, Minh BQ. 2015. IQ-TREE: a fast and effective stochastic algorithm for estimating maximum-likelihood phylogenies. Mol Biol Evol. 32(1):268–274. 25371430 10.1093/molbev/msu300 PMC4271533

[evab265-B53] Nunes VS , Damasceno JD, Freire R, Tosi LRO. 2011. The Hus1 homologue of *Leishmania* major encodes a nuclear protein that participates in DNA damage response. Mol Biochem Parasitol. 177(1):65–69. 21291918 10.1016/j.molbiopara.2011.01.011

[evab265-B54] Nygaard S , et al 2010. Long- and short-term selective forces on malaria parasite genomes. PLoS Genet. 6(9):e1001099. 20838588 10.1371/journal.pgen.1001099 PMC2936524

[evab265-B55] Ochola-Oyier L , Isabella, et al 2019. Few *Plasmodium falciparum* merozoite ligand and erythrocyte receptor pairs show evidence of balancing selection. Infect Genet Evol. 69:235–245. 30735814 10.1016/j.meegid.2019.02.004 PMC6403450

[evab265-B56] Purcell S , et al 2007. PLINK: a tool set for whole-genome association and population-based linkage analyses. Am J Hum Genet. 81(3):559–575. 17701901 10.1086/519795 PMC1950838

[evab265-B57] Quinlan AR , Hall IM. 2010. BEDTools: a flexible suite of utilities for comparing genomic features. Bioinformatics 26(6):841–842. 20110278 10.1093/bioinformatics/btq033 PMC2832824

[evab265-B1283333] Renaud G. 2018. glactools: a command-line toolset for the management of genotype likelihoods and allele counts. Bioinformatics 34(8):1398–1400. 29186325 10.1093/bioinformatics/btx749

[evab265-B58] Rocker A , Meinhart A. 2015. A Cis-acting antitoxin domain within the chromosomal toxin–antitoxin module EzeT of *Escherichia coli* quenches toxin activity. Mol Microbiol. 97(3):589–604. 25943309 10.1111/mmi.13051

[evab265-B59] Rogers Matthew B , et al 2014. Genomic confirmation of hybridisation and recent inbreeding in a vector-isolated *Leishmania* population. PLoS Genet. 10(1):e1004092. 24453988 10.1371/journal.pgen.1004092 PMC3894156

[evab265-B60] Schwabl P , et al 2021. Colonization and genetic diversification processes of *Leishmania infantum* in the Americas. Commun Biol. 4(1):139. 33514858 10.1038/s42003-021-01658-5 PMC7846609

[evab265-B61] Sellis D , Callahan BJ, Petrov DA, Messer PW. 2011. Heterozygote advantage as a natural consequence of adaptation in diploids. Proc Natl Acad Sci U S A. 108(51):20666–20671. 22143780 10.1073/pnas.1114573108 PMC3251125

[evab265-B57517797] Siewert KM , Voight BF. 2020. BetaScan2: Standardized statistics to detect balancing selection utilizing substitution data. Genome Biol Evol. 12(2):3873–3877. 32011695 10.1093/gbe/evaa013 PMC7058154

[evab265-B62] Siewert KM , Voight BF. 2017. Detecting long-term balancing selection using allele frequency correlation. Mol Biol Evol. 34(11):2996–3005. 28981714 10.1093/molbev/msx209 PMC5850717

[evab265-B63] Srivastava A , et al 2019. Identification and functional characterization of a bacterial homologue of zeta toxin in *Leishmania donovani*. FEBS Lett. 593(11):1223–1235. 31074836 10.1002/1873-3468.13429

[evab265-B64] Sunter JD , et al 2015. Modulation of flagellum attachment zone protein FLAM3 and regulation of the cell shape in *Trypanosoma brucei* life cycle transitions. J Cell Sci. 128(16):3117–3130. 26148511 10.1242/jcs.171645 PMC4541047

[evab265-B65] Sunter JD , et al 2019. *Leishmania flagellum* attachment zone is critical for flagellar pocket shape, development in the sand fly, and pathogenicity in the host. Proc Natl Acad Sci U S A. 116(13):6351–6360. 30850532 10.1073/pnas.1812462116 PMC6442623

[evab265-B66] Tajima F. 1989. Statistical method for testing the neutral mutation hypothesis by DNA polymorphism. Genetics 123 (3):585–595. 2513255 10.1093/genetics/123.3.585 PMC1203831

[evab265-B67] Teixeira DG , et al 2017. Comparative analyses of whole genome sequences of *Leishmania infantum* isolates from humans and dogs in Northeastern Brazil. Int J Parasitol. 47(10–11):655–665. 28606698 10.1016/j.ijpara.2017.04.004 PMC5641220

[evab265-B68] Tetteh Kevin KA , et al 2009. Prospective identification of malaria parasite genes under balancing selection. PLoS One. 4(5):e5568. 19440377 10.1371/journal.pone.0005568 PMC2679211

[evab265-B69] Thakur CP. 2007. A new strategy for elimination of Kala-Azar from rural Bihar. Indian J Med Res. 126(5):447–451. 18160749

[evab265-B70] Volkman SK , et al 2002. Excess polymorphisms in genes for membrane proteins in *Plasmodium falciparum*. Science 298(5591):216–218. 12364807 10.1126/science.1075642

[evab265-B71] Wahlgren M , Goel S, Akhouri RR. 2017. Variant surface antigens of *Plasmodium falciparum* and their roles in severe malaria. Nat Rev Microbiol. 15(8):479–491. 28603279 10.1038/nrmicro.2017.47

[evab265-B72] Walker BJ , et al 2014. Pilon: an integrated tool for comprehensive microbial variant detection and genome assembly improvement. PLoS One 9(11):e112963. 25409509 10.1371/journal.pone.0112963 PMC4237348

[evab265-B73] Wang M , et al 2020. Phylogenomics of the genus populus reveals extensive interspecific gene flow and balancing selection. New Phytol. 225(3):1370–1382. 31550399 10.1111/nph.16215

[evab265-B74] Weedall GD , Conway DJ. 2010. Detecting signatures of balancing selection to identify targets of anti-parasite immunity. Trends Parasitol. 26(7):363–369. 20466591 10.1016/j.pt.2010.04.002

[evab265-B75] Westram AM , Faria R, Johannesson K, Butlin R. 2021. Using replicate hybrid zones to understand the genomic basis of adaptive divergence. Mol Ecol. 30(15):3797–3814. 33638231 10.1111/mec.15861

[evab265-B76] Wood HM , Grahame JW, Humphray S, Rogers J, Butlin RK. 2008. Sequence differentiation in regions identified by a genome scan for local adaptation. Mol Ecol. 17(13):3123–3135. 18510587 10.1111/j.1365-294X.2008.03755.x

[evab265-B77] World Health Organisation. 2020. Leishmaniasis. [cited 2020 Mar 2]. Available from: https://www.who.int/news-room/fact-sheets/detail/leishmaniasis

[evab265-B78] Zackay A , et al 2018. Genome wide comparison of Ethiopian *Leishmania donovani* strains reveals differences potentially related to parasite survival. PLoS Genet. 14(1):e1007133. 29315303 10.1371/journal.pgen.1007133 PMC5777657

[evab265-B79] Zhang WW , et al 2014. Genetic analysis of *Leishmania donovani* tropism using a naturally attenuated cutaneous strain. PLoS Pathog. 10(7):e1004244. 24992200 10.1371/journal.ppat.1004244 PMC4081786

