# Supplementary material for: Candidates for Balancing Selection in Leishmania donovani Complex Parasites
Source: Genome Biol Evol. 2021 Dec 2;13(12):evab265. doi: 10.1093/gbe/evab265 (PMC8717319; doi:10.1093/gbe/evab265)
Supplement: evab265_Supplementary_Data [file evab265_supplementary_data.zip › Clean Copy of Supplementary Material-2021-12-10.docx]

# **Supplementary Text**

## **Supplementary Text 1. Duplications distort allele frequencies**

Initial analysis of the allele frequencies of these populations showed a tail of high MAF alleles that would be consistent with strong balancing selection in some sites. However, closer inspection of these data showed that all populations had a strong positive correlation between read coverage and MAF (**Supplementary Figure 5**). Very high copy regions, which included multiple duplicated genes, were significantly enriched for common alleles. This bias is most likely due to systematic under-calling of rare alleles in duplicated genes, combined with the appearance of ‘balanced’ allele frequencies caused by variants fixed in one duplication (**Supplementary Figure 15**). These processes will distort allele frequencies, and result in artefacts in many of the methods used to detect balancing selection, such as Tajima’s *D* [(Tajima 1989)](https://paperpile.com/c/6meJey/JhKm5) and *NCD2* [(Bitarello et al. 2018)](https://paperpile.com/c/6meJey/aflp3), and *BetaScan*/Betascan2* which utilise correlated alleles [(Siewert and Voight 2017, 2020)](https://paperpile.com/c/6meJey/D3DmX+5n1ip). As the missing rare alleles are generally newer, this process will also reduce population distances (F_ST_), an expectation of long term balancing selection [(Charlesworth 2006)](https://paperpile.com/c/6meJey/8f30K).

##

## **Supplementary Text 2. Full description of BS tests and justifications**

To identify the most likely candidates of BS in these populations we applied a search strategy that used genomic window analyses, followed by gene-centric analysis (**Supplementary Figure 5**). To identify genomic windows that contain signals consistent with BS we applied two tests, *Betascan** [(Siewert and Voight 2017)](https://paperpile.com/c/6meJey/D3DmX) and *NCD2* [*(Bitarello et al. 2018)*](https://paperpile.com/c/6meJey/aflp3) over 10 kb windows around each segregating site, for each population. *Betascan** detects regions with alleles ‘balanced’ at correlated frequencies and a deficit of substitutions compared to the outgroup, while the *NCD2* test detects regions with an excess of alleles near a target frequency (0.5 in our case). Selection of outlier regions with *Betascan** and/or *NCD2* scores in any population identified 258 genes (13 were identified in more than one population).

For gene-centric analysis, we identified specific genes that may be BS targets within these windows, therefore we calculated nucleotide diversity (π) and Tajima’s *D* [(Tajima 1989)](https://paperpile.com/c/6meJey/JhKm5) for all genes in all populations (**Supplementary Table 5**). We selected genes in the 90th percentile of either statistic for any population as the most likely targets of balancing selection. Neither of these gene sets were significantly enriched for any Gene Ontology categories after multiple-test correction. Selection of genes with both genomic window tests (*NCD2* or *Betascan*) and gene-centric metrics (Tajima’s *D* or π) identified 33 genes (**Supplementary Table 4**).

#

# **Supplementary Figures**


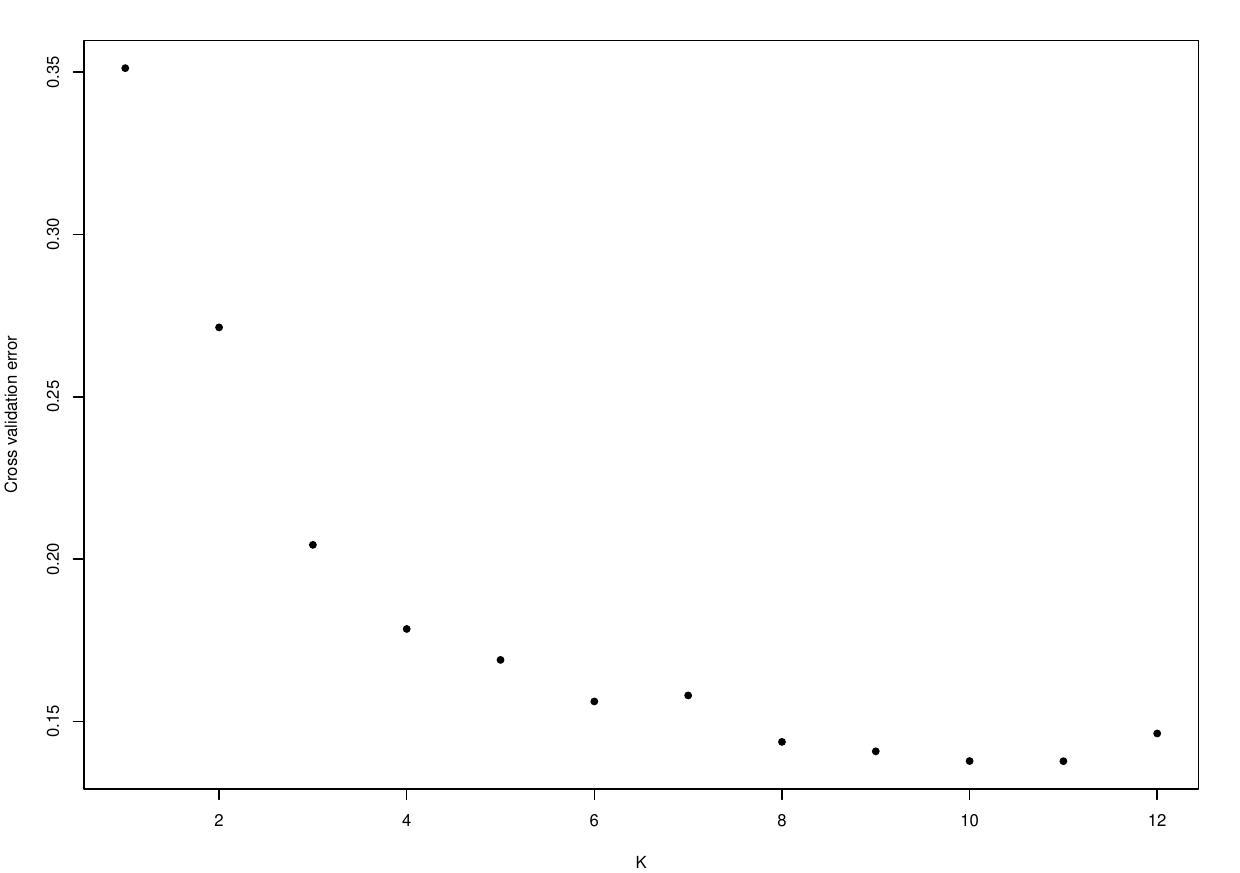


**Supplementary Figure 1. ADMIXTURE cross-validation error for *K*=1-12.** ADMIXTURE analysis of 477 isolates indicated a true population number between 8-11.


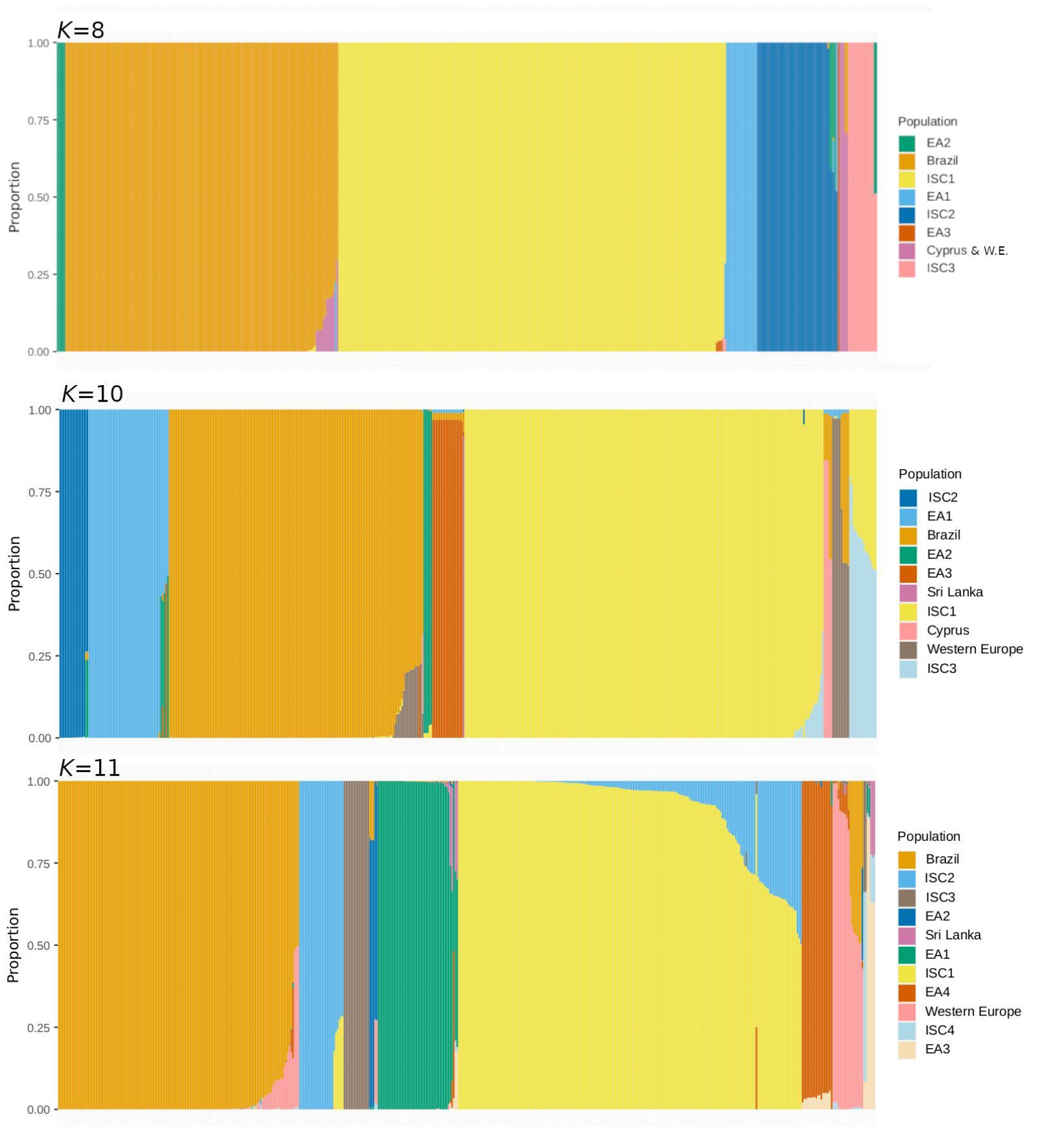


**Supplementary Figure 2. ADMIXTURE results for *K*=8, 10 and 11.** Isolates assigned to populations when *K*= 8, 10 and 11. Each vertical bar represents a single isolate. We proceeded with *K*=9 (**Figure 1**) in line with previous analysis by [(Franssen et al. 2020)](https://paperpile.com/c/6meJey/uQADf). Populations are named according to their predominant origin. WE = Western Europe.


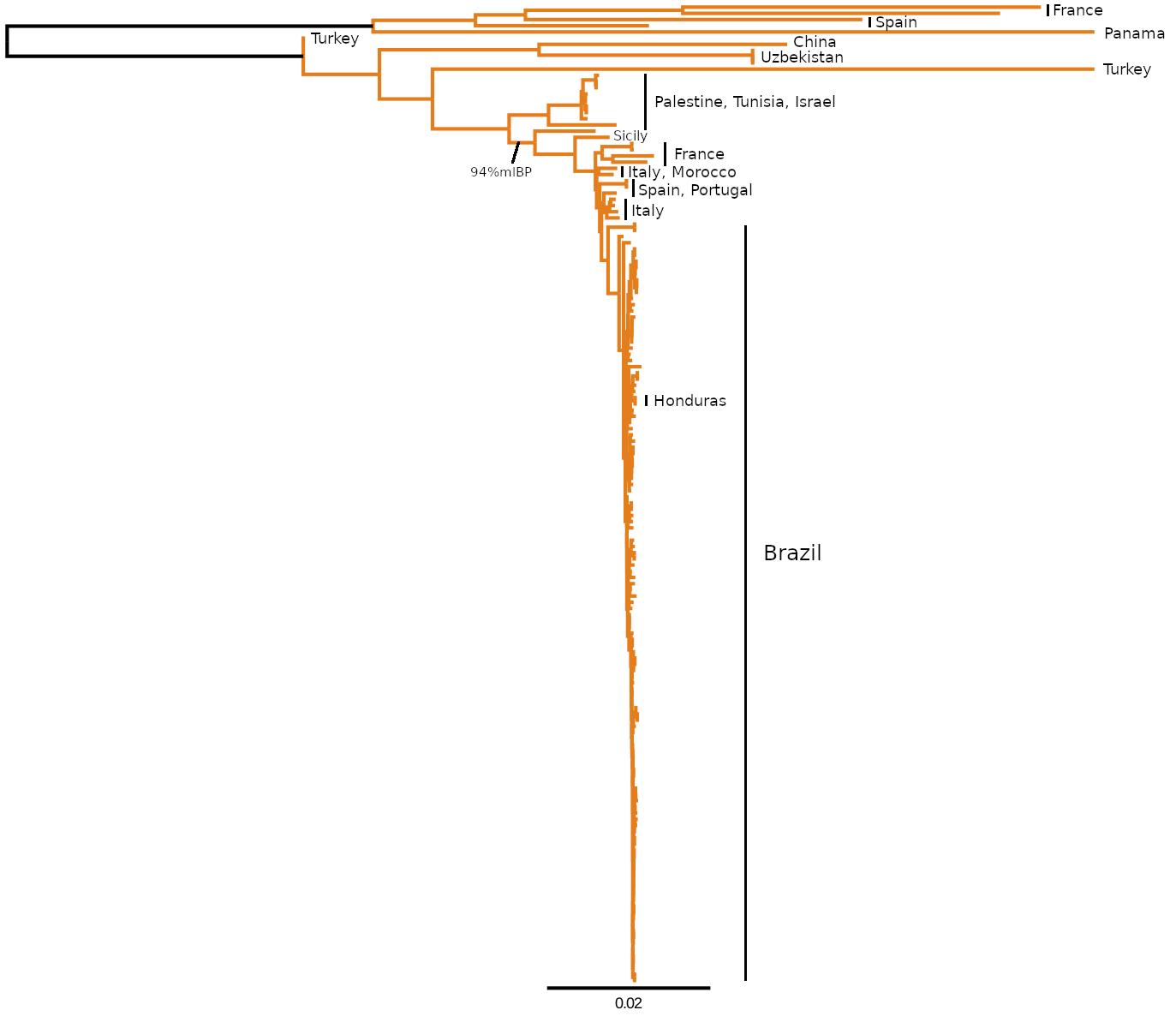


**Supplementary Figure 3. Maximum likelihood phylogeny of BM isolates.** Tree of *L. infantum* strains, based upon a SNP alignment of 158 sequences with 81,018 variable sites, midpoint rooted. 93 of these isolates were sequenced in the current work. Countries of isolate origin are used as tip names. All visible branches are maximally supported (100% mlBP) unless indicated. The scale bar represents the number of nucleotide changes per site.


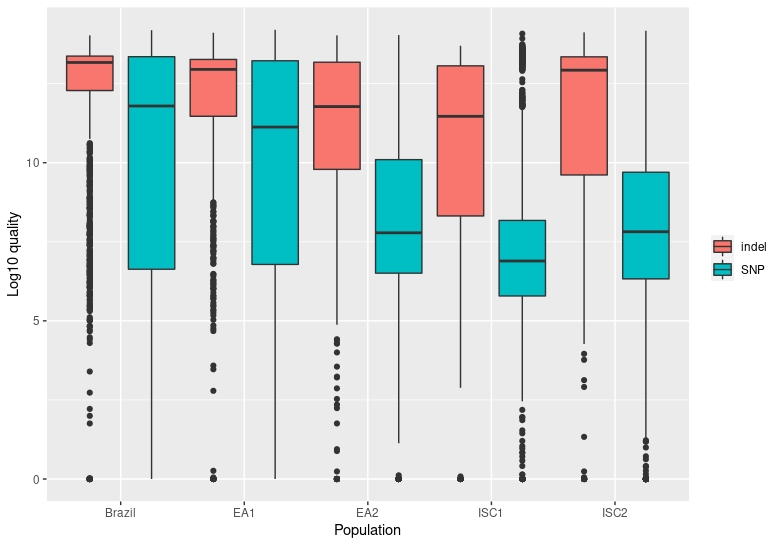


**Supplementary Figure 4.** **The excess of indels is unlikely to be artefactual.** Log10 QUAL scores for SNP and indel positions are displayed for each population.


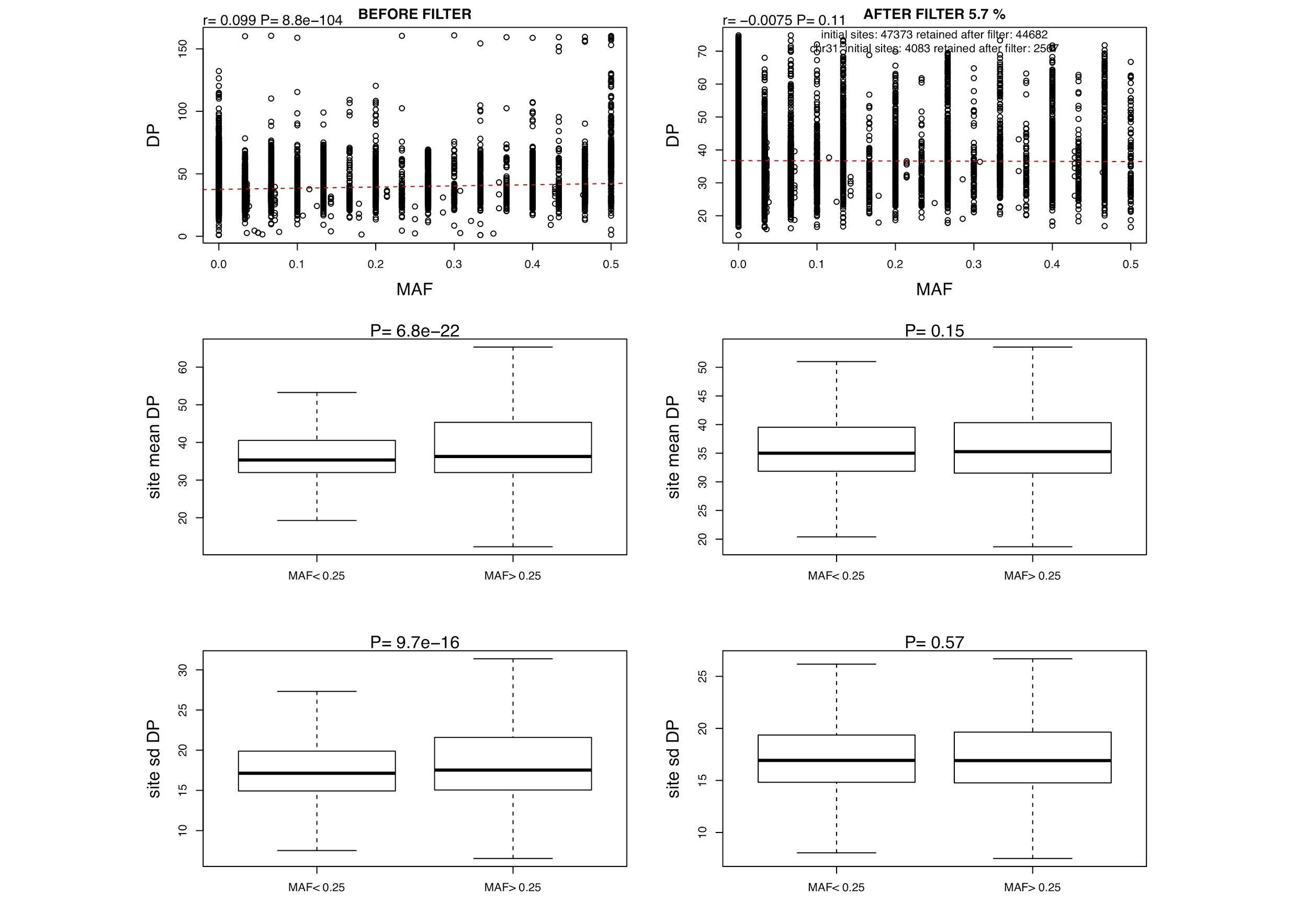


**Supplementary Figure 5. Duplications systematically bias allele frequencies.** Data shown are from the ISC2 population. Left three panels show data prior to depth filtering. Top panel: a positive correlation between total read depth (DP) and minor allele frequency (MAF). Middle panel: sites with MAF > 0.25 are significantly higher read depth. Lower panel: sites with MAF > 0.25 are significantly higher variation in (standard deviation, sd) in read depth. Right three panels show the same presentations of data after read depth filtering. None of the relationships remain statistically significant.


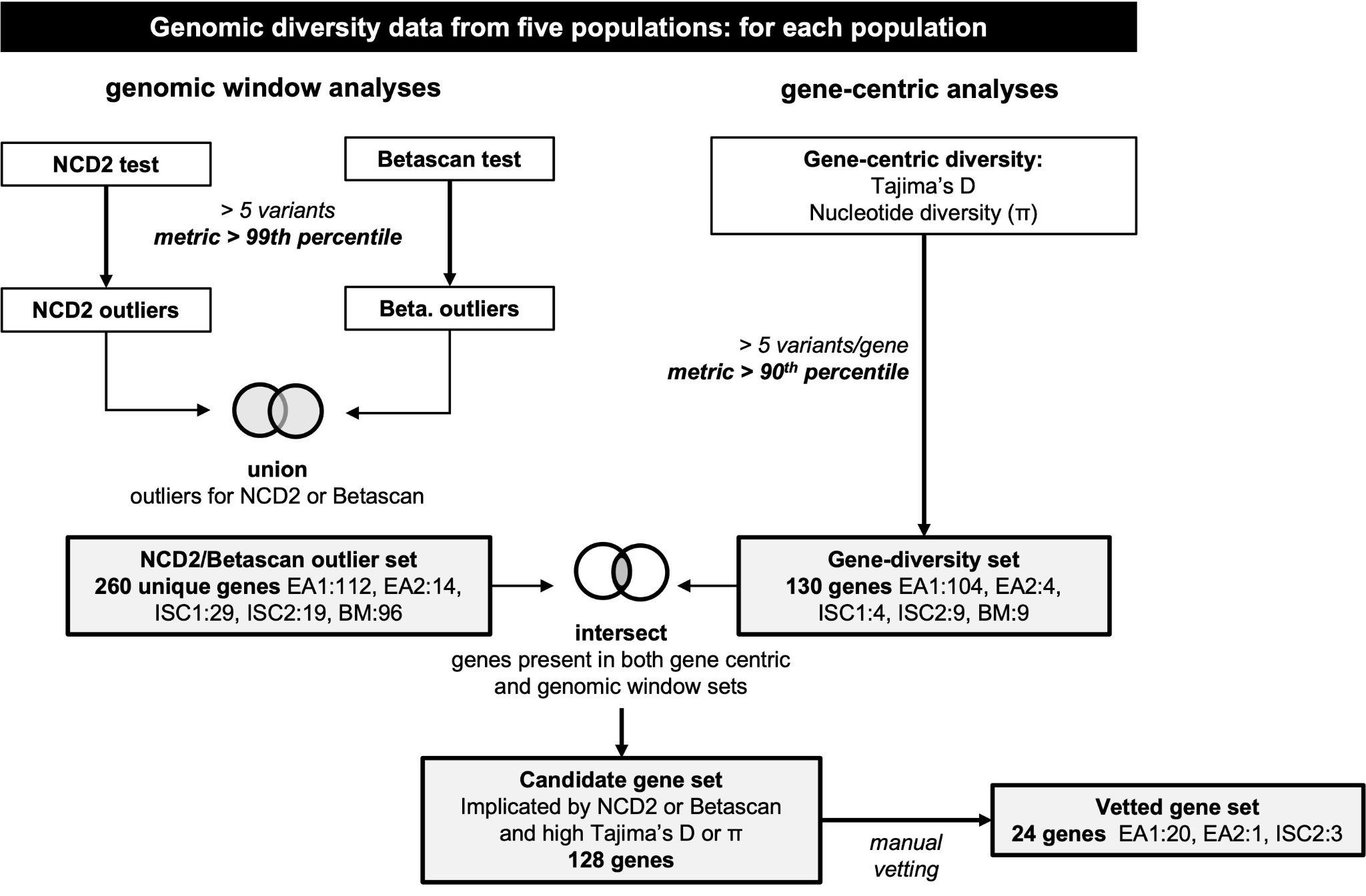


**Supplementary Figure 6. Search strategy employed in balancing selection search**. Full description in **Supplementary Text 1**.

**
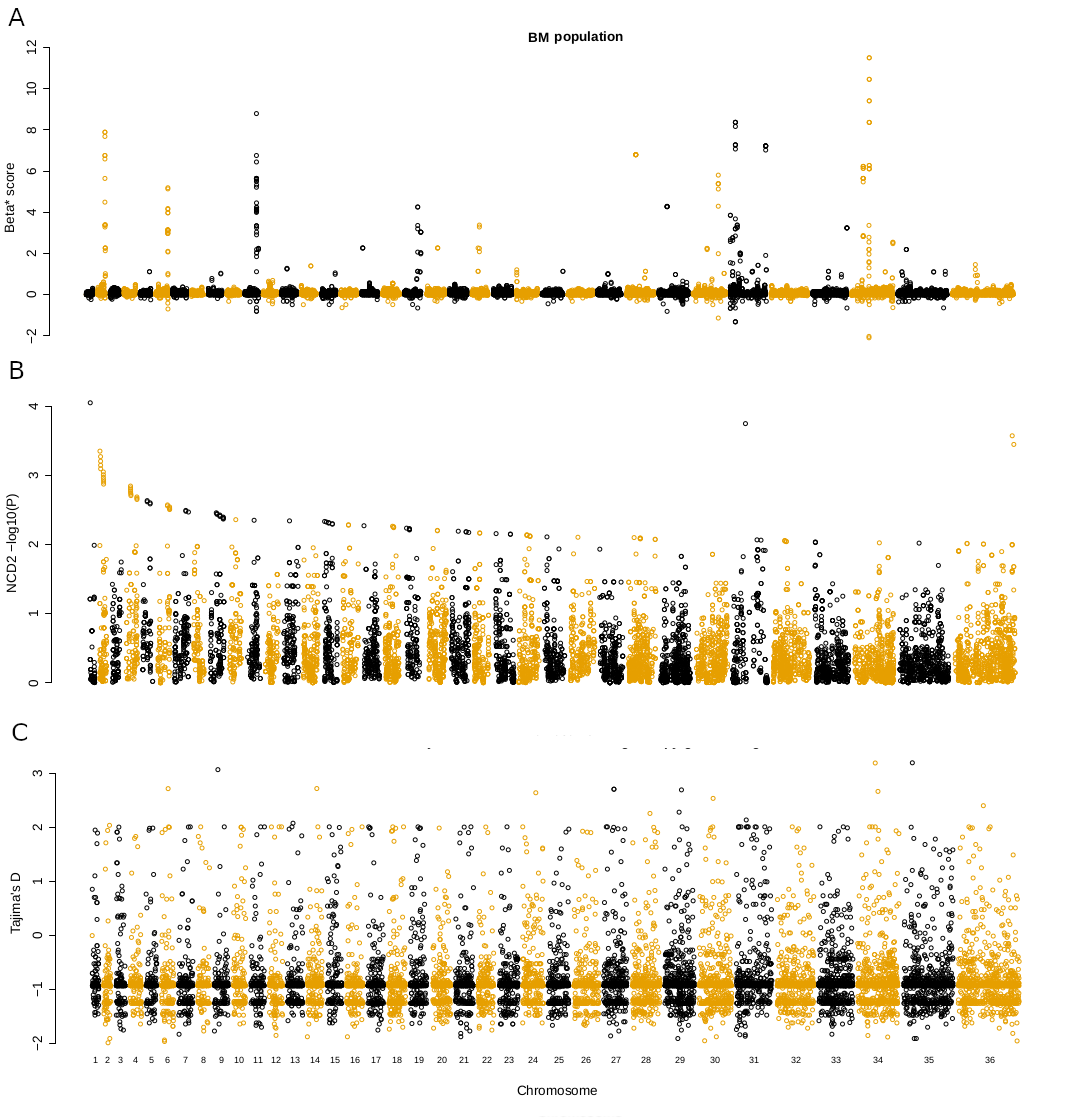
**

**Supplementary Figure 7. Comparison of balancing selection tests for population BM. Panel A**: Betascan*; **Panel B**: NCD2 -log10 P values; **Panel C**: Tajima’s *D*. For each test, values obtained in 10kb windows are plotted for each chromosome.

**
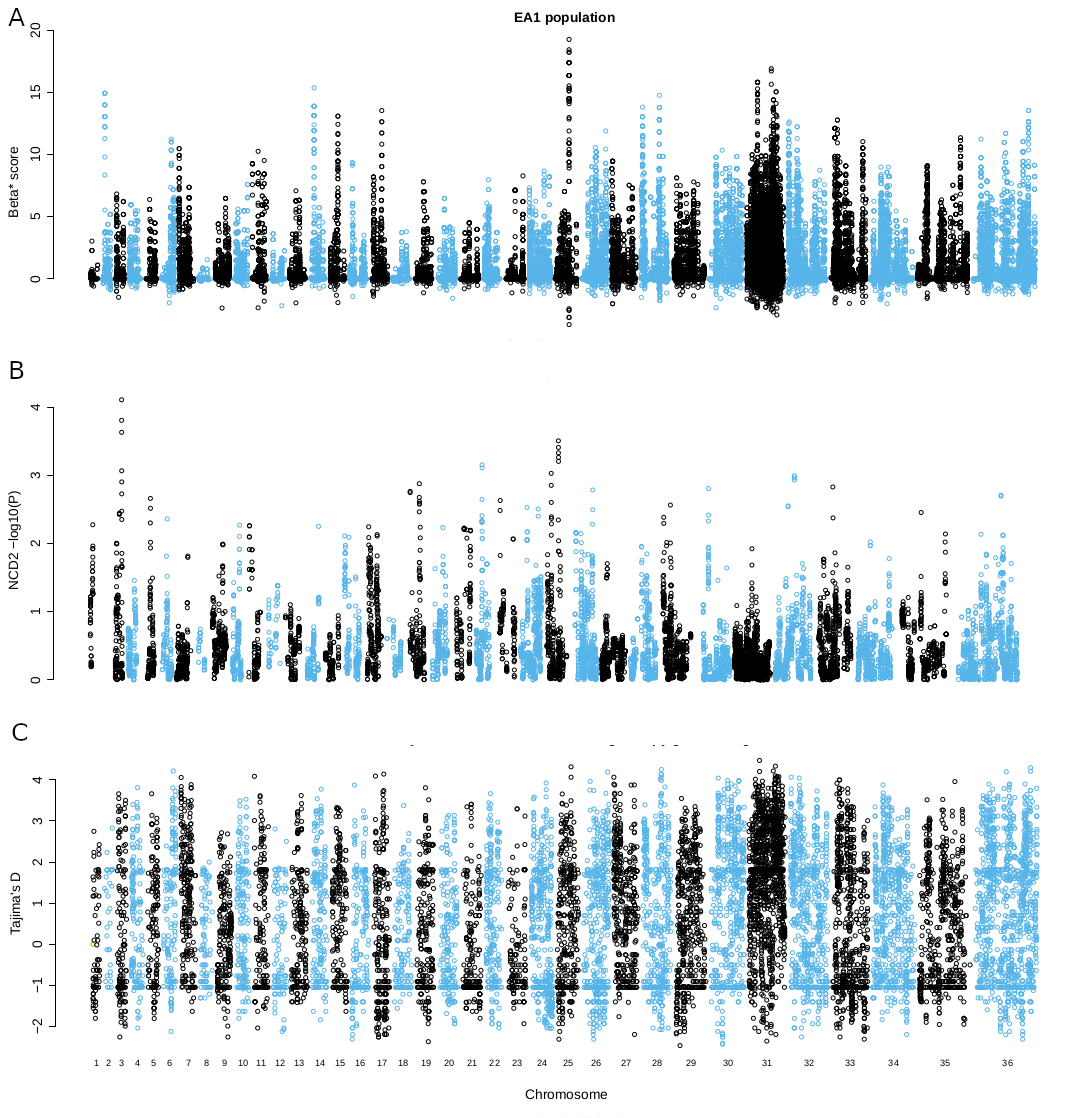
**

**Supplementary Figure 8. Comparison of balancing selection tests for population EA1. Panel A**: Betascan* **Panel B**: NCD2 -log10 P values; **Panel C**: Tajima’s *D*. For each test, values obtained in 10kb windows are plotted for each chromosome.

**
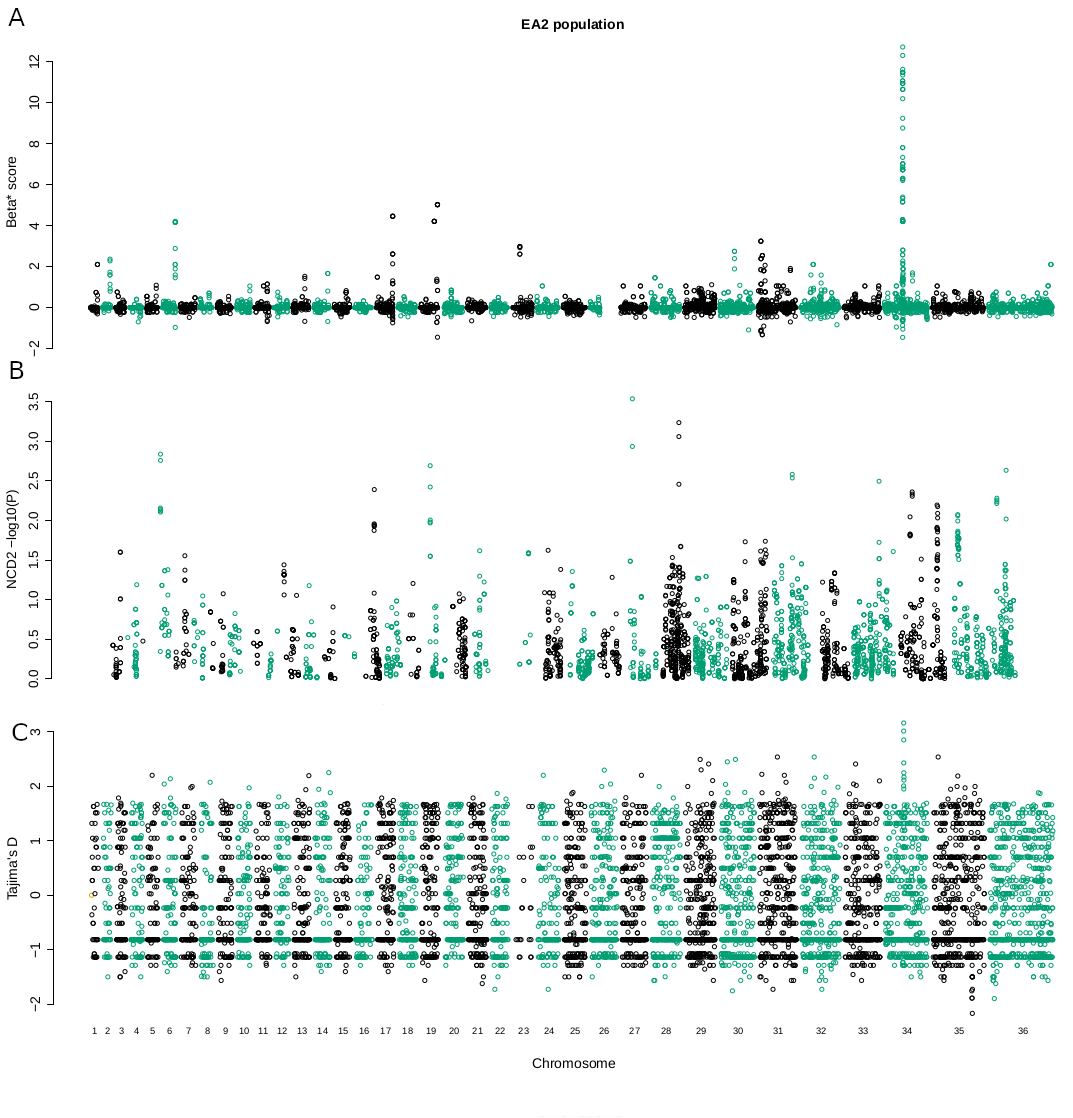
**

**Supplementary Figure 9. Comparison of balancing selection tests for population EA2. Panel A**: Betascan* **Panel B**: NCD2 -log10 P values; **Panel C**: Tajima’s *D*. For each test, values obtained in 10kb windows are plotted for each chromosome.


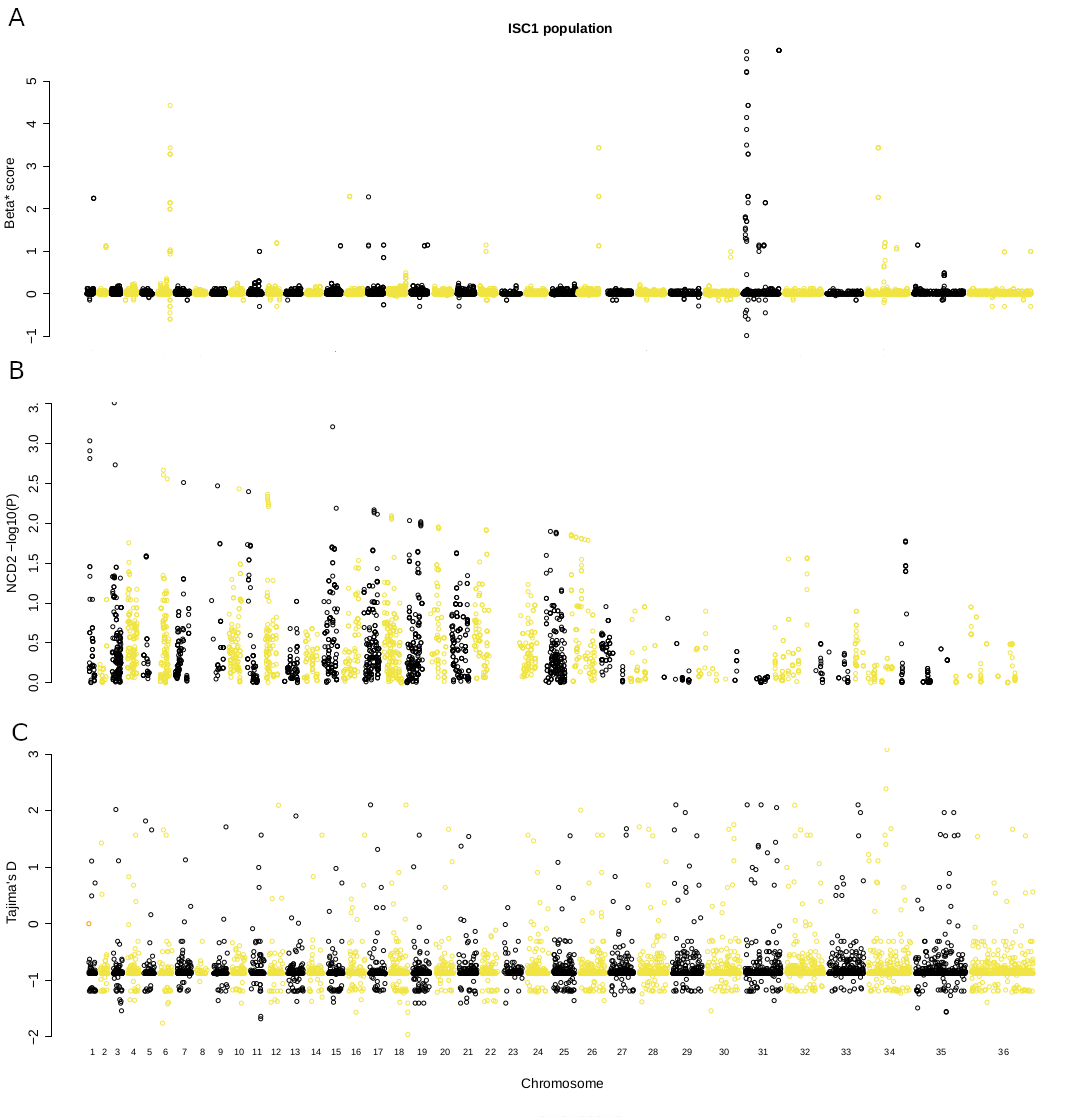


**Supplementary Figure 10. Comparison of balancing selection tests for population ISC1. Panel A**: Betascan* **Panel B**: NCD2 -log10 P values; **Panel C**: Tajima’s *D*. For each test, values obtained in 10kb windows are plotted for each chromosome.

**
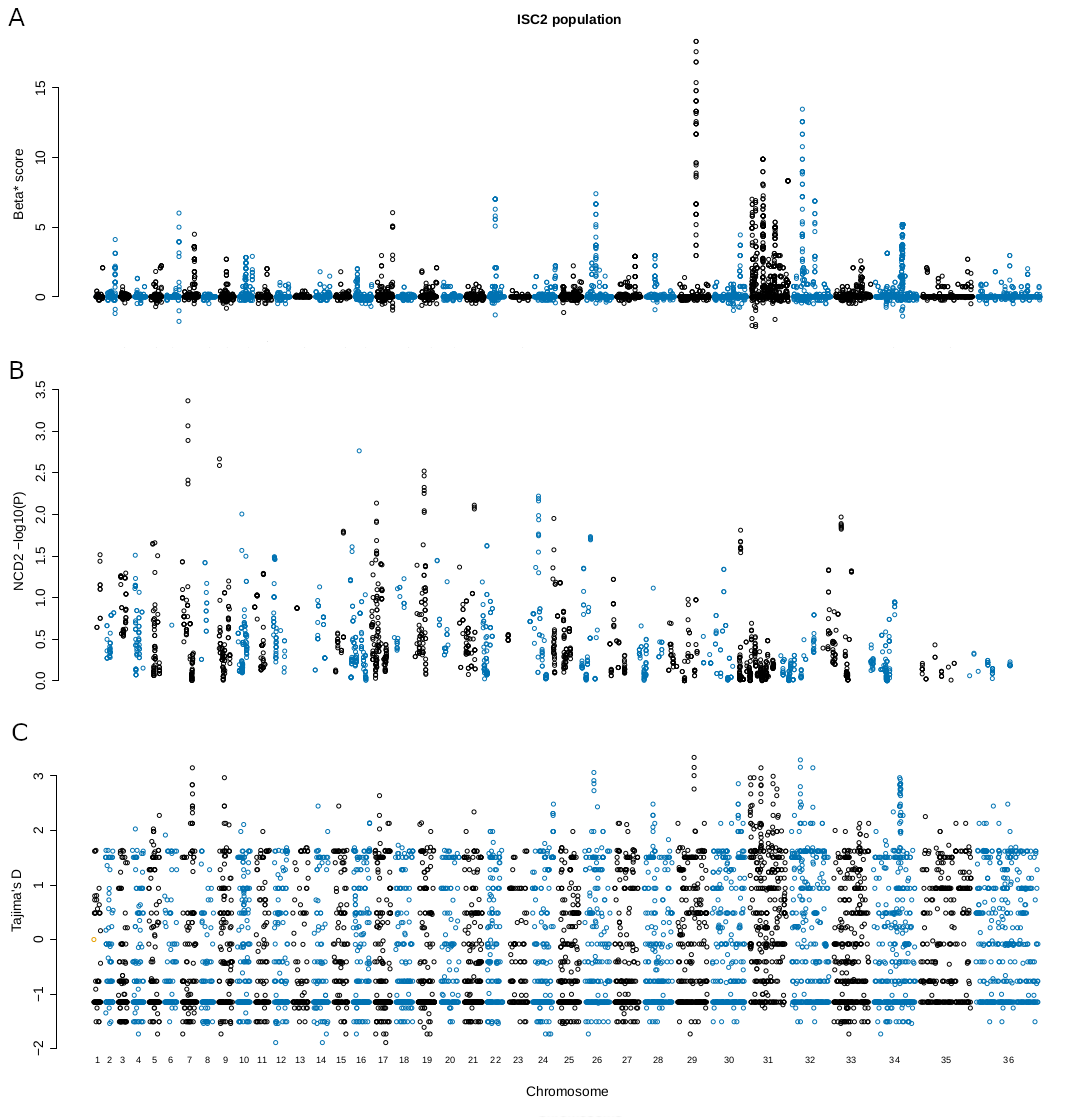
**

**Supplementary Figure 11. Comparison of balancing selection tests for population ISC2. Panel A**: Betascan*; **Panel B:** NCD2 -log10 P values; **Panel C**: Tajima’s *D*. For each test, values obtained in 10kb windows are plotted for each chromosome.


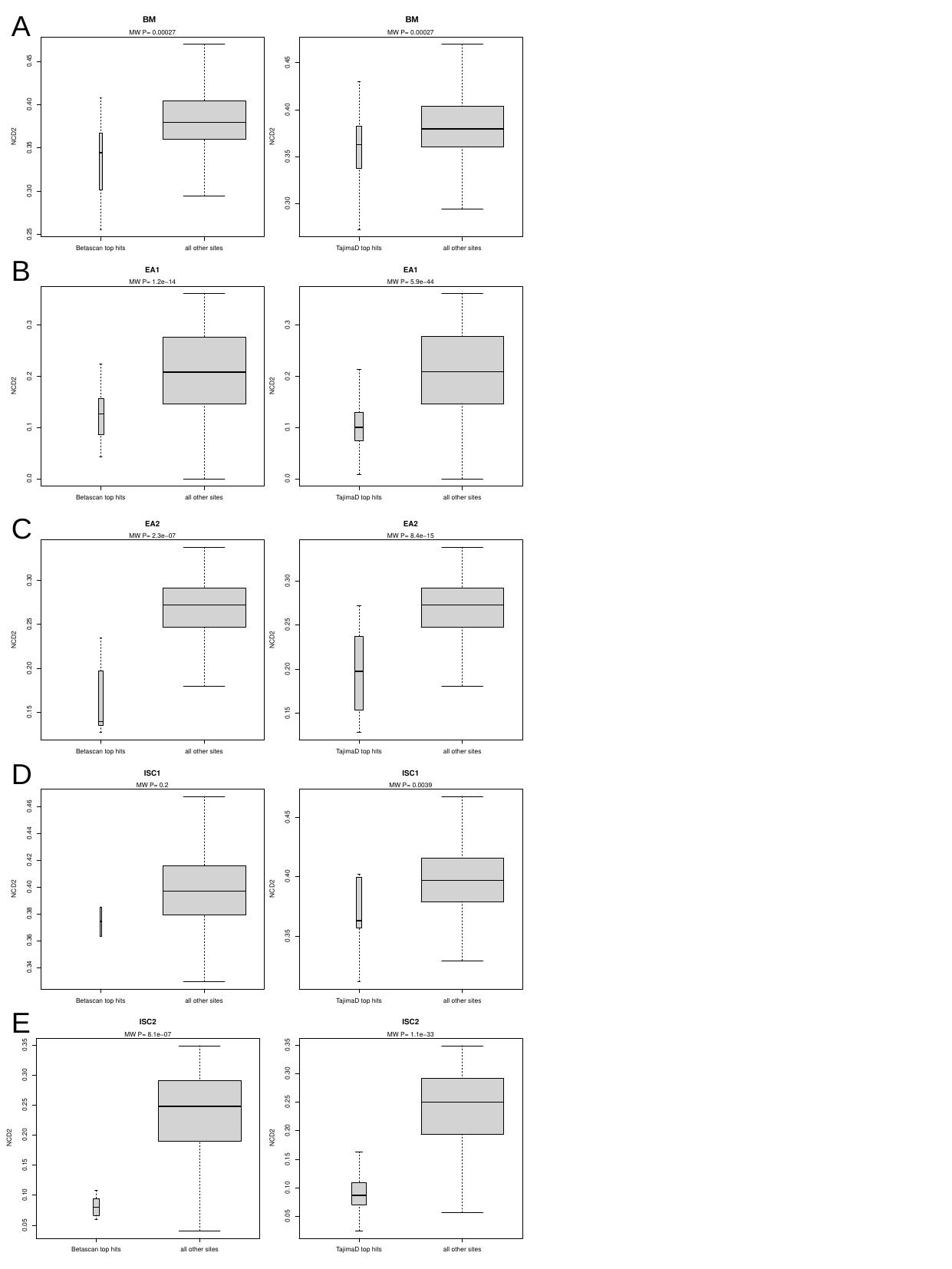


**Supplementary Figure 12. Consistency between tests for balancing selection.** In each population, *Betascan* outliers in the 99th percentile of the 𝛽 statistic [(Siewert and Voight 2017)](https://paperpile.com/c/6meJey/D3DmX) were enriched for low *NCD2* scores(left column) values, and high Tajima's *D* (right column). Note that *NCD2* scores are expected to be *low* for sites subject to BS [(Bitarello et al. 2018)](https://paperpile.com/c/6meJey/aflp3), while Tajima's *D* will return *higher* values for BS sites. **Panel** **A** - BM; **B**- EA1; **C** - EA2; **D** - ISC1; **E** - ISC2.

**
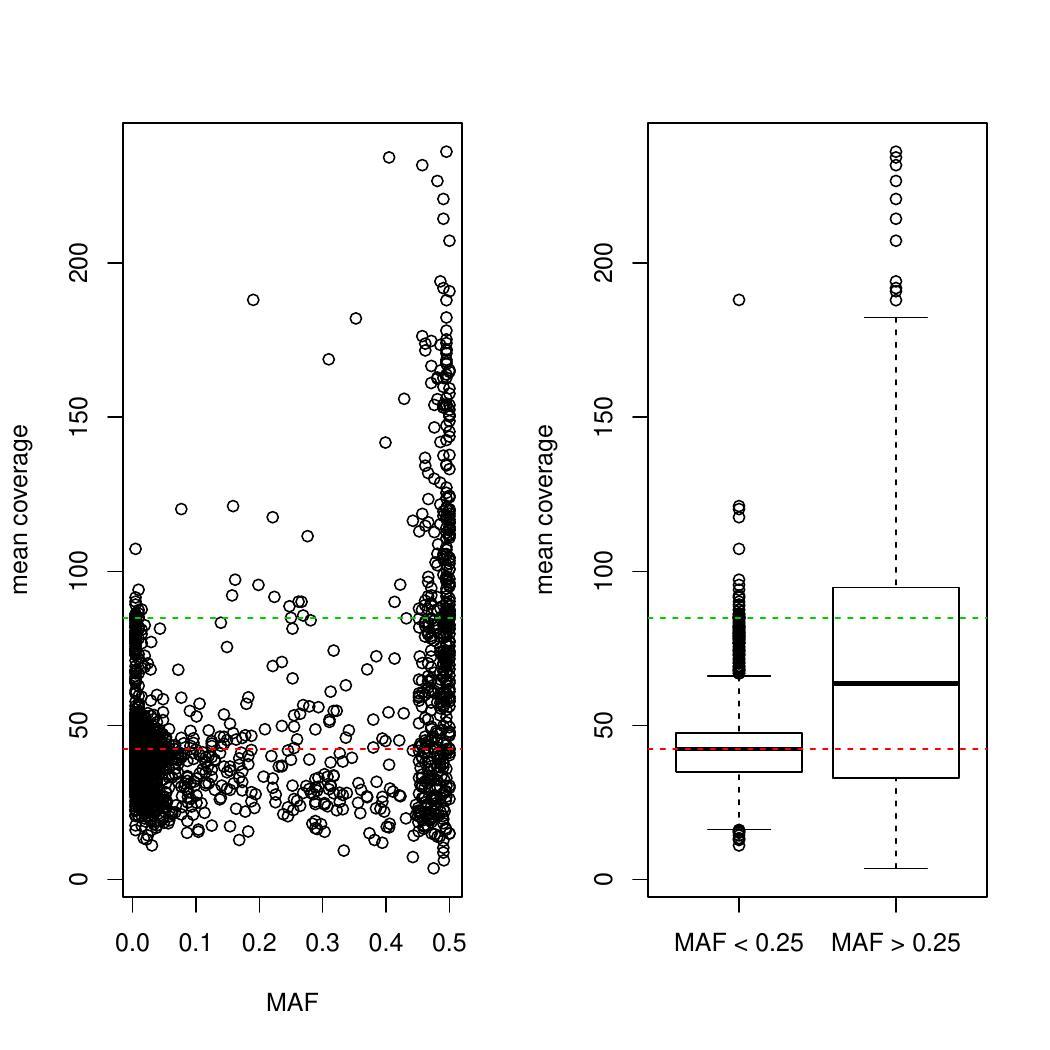
**

**Supplementary Figure 13.** Correlation between MAF and read coverage at SNP sites. Modelling showed that duplications resulted in a systematic bias against calling rare alleles. We removed any SNP/indel sites where the mean variant coverage within the ADMIXTURE-defined population was ≥1.5x larger than the median coverage (corresponding to triploid sites in a generally diploid chromosome), or ≥1.25x larger than the median coverage for chromosome 31 (corresponding to tetraploid sites in a generally triploid chromosome). We also removed sites where coverage was highly variable, by excluding sites in the upper 5th percentile of the standard deviation.

**
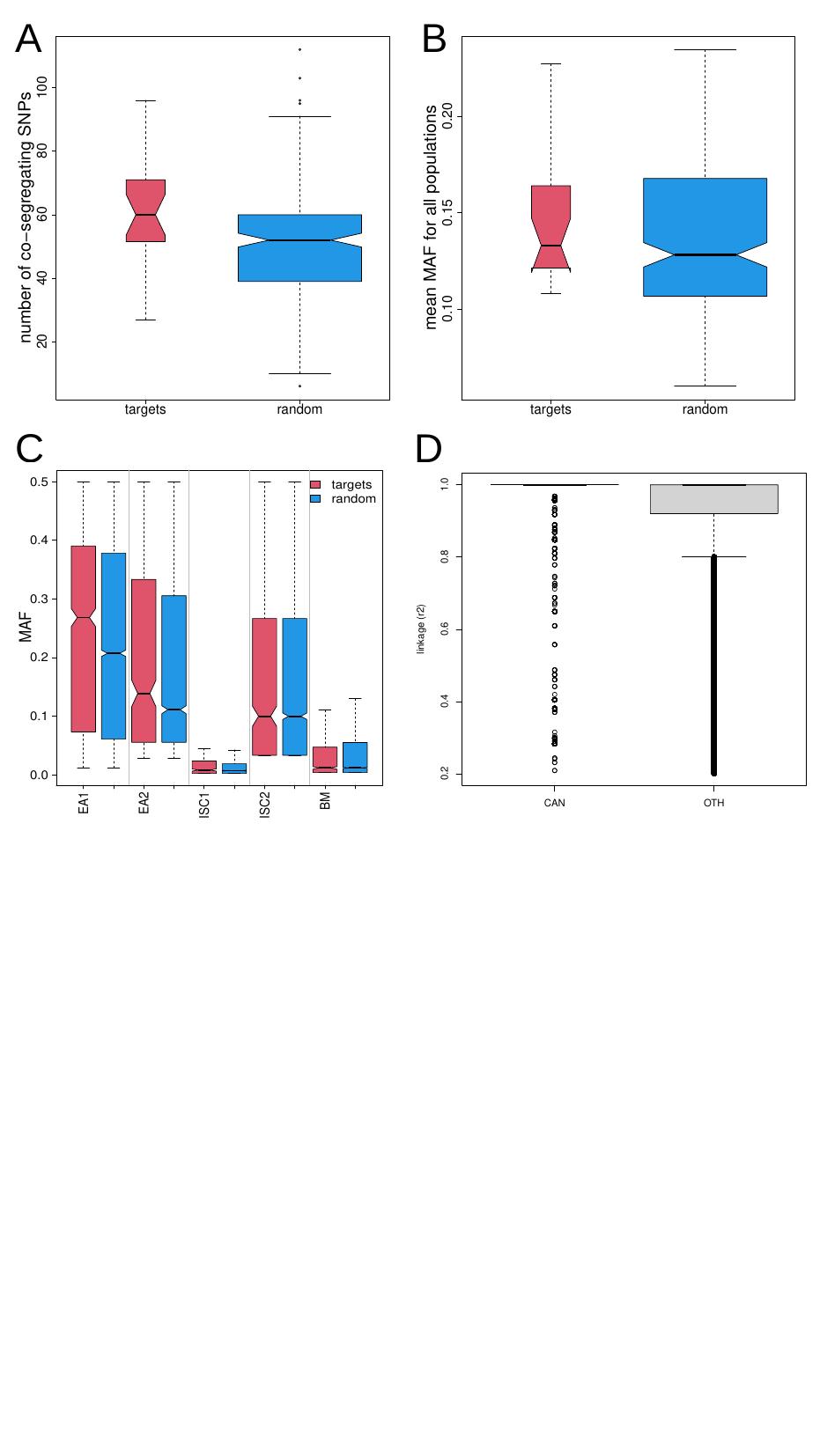
**

**Supplementary Figure 14. Candidates for BS are enriched for high MAF co-segregating SNPs. Panel A**: for the 24 validated candidate BS genes, we show the number of variants (SNPs/indels) in 130 kb proximal genomic windows that segregate between more than one population, compared to 330 length- and chromosome-matched genomic windows chosen at random. These distributions are significantly different (Mann-Whitney *P* = 0.008122). **Panel B**: these variants do not have higher mean minor allele frequencies (MAF, means calculated between all populations) compared to matched random controls (Mann-Whitney *P* = 0.1095). **Panel C**: comparison of the MAF of co-segregating SNPs between populations indicates that target MAF is elevated in the East African populations EA1 (where most targets were discovered) and EA2. **Panel D**: the 20 validated BS candidates in EA1 show higher levels of statistical linkage than genome-wide distributions. Left box (CAN) is linkage in the candidates; right box (OTH) is all other sites. This is consistent with expectations for BS, as candidate genes have higher linkage (Wilcox test P = 4.037796e-28).


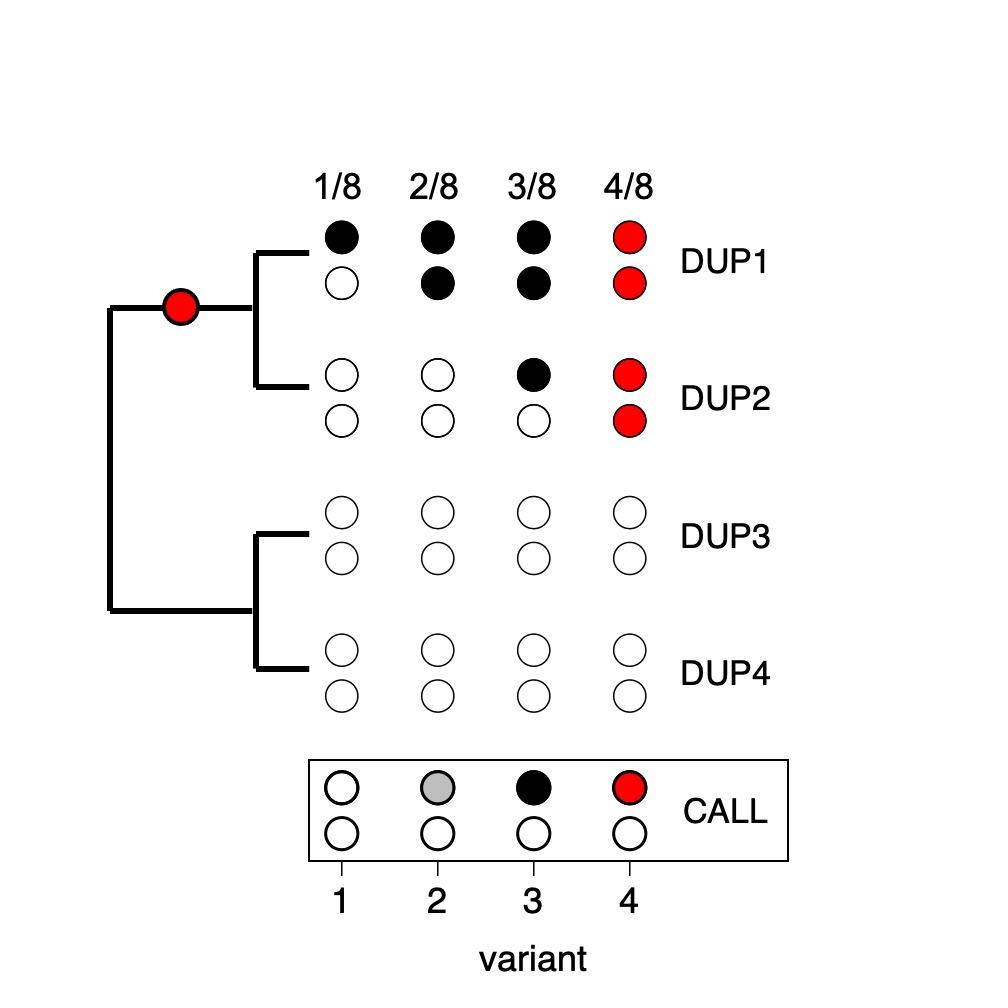


**Supplementary Figure 15. Systematic under-calling of rare alleles in duplicated genes.** We consider variant calling in one strain, in a gene that contains four copies, and four variant positions. Duplications have arisen with some arbitrary phylogeny. In a diploid chromosome, there are eight haplotypes. The likely consensus variant call by a variant-caller that assumes a single diploid site is shown in the box below. *Loss of rare alleles.* Rare alleles, such as singletons that have arisen in one copy (variant 1) will be represented by ⅛th of the reads, so are unlikely to be called (two open dots in consensus call). More common alleles, such as those homozygous in one duplication (variant 2), may or not be called, depending on details of variant calling, and stochastic read counts for the alleles (grey dot in consensus call). More common variants in duplications are increasingly likely to be called as heterozygous sites in the consensus call (variant 3). *Appearance of balanced alleles.* In the rare case that a variant becomes fixed in one clade of the duplication phylogeny (variant 4, and red dot in phylogeny), it will be called as a heterozygous site. Depending on the age of the duplication, such a site may be called as a heterozygous site in many/all strains. This will produce the appearance of a balanced polymorphism.

**Supplementary Figure 16. Vignettes of all candidate genes.** Panels from top: Betascan*, NCD1, Tajima’s *D*, nucleotide diversity, MAF and coverage. Note to reviewers: this figure has been uploaded to FigShare, split by population.

# **
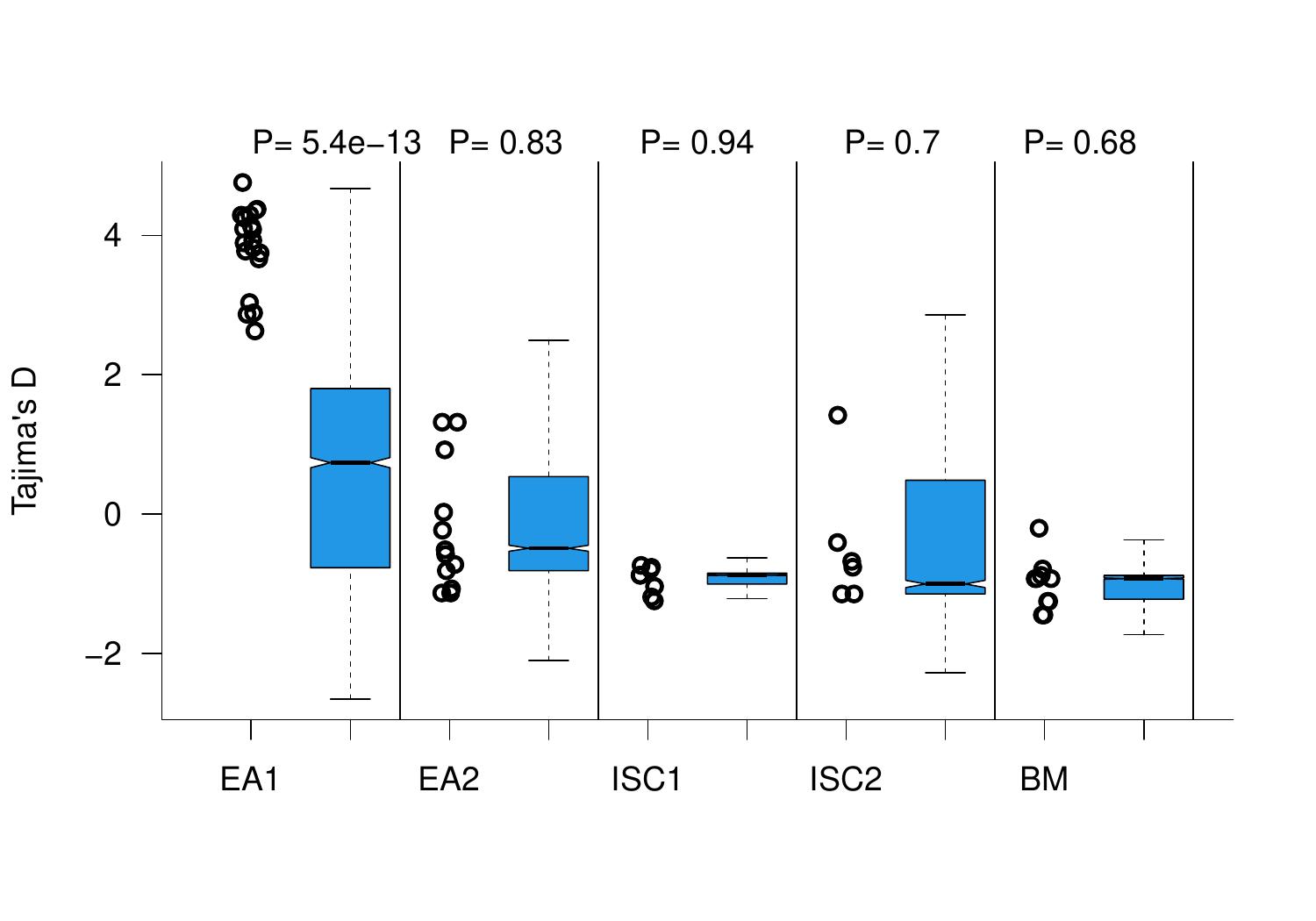
**

**Supplementary Figure 17.** For the 24 candidate BS genes that were discovered in the EA1 population, we show the Tajima’s *D* values in all populations (open circles), relative to the genome-wide distribution (blue boxes). All p-values are Mann-Whitney tests of candidate BS genes compared to all other genes. This suggests that BS signals are generally not consistent between populations (see also **Supplementary Figure 18**).


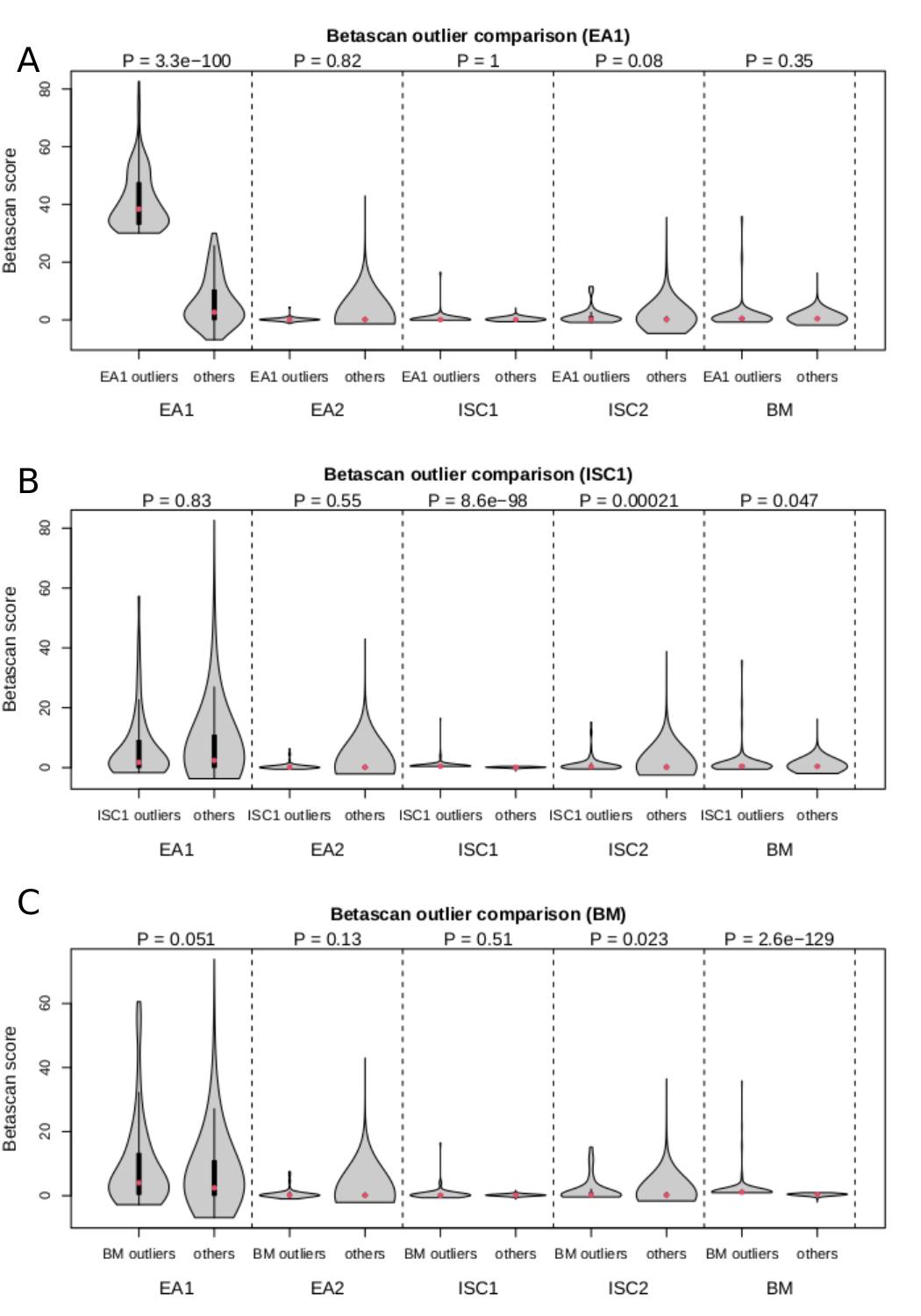


**Supplementary Figure 18. BS signals from Betascan are generally not consistent between populations.** For each of three populations (EA1, ISC1, BM), we identify the 5% outlier genes for the *Betascan** metric. We then examine whether these genes have significantly higher *Betascan** scores in any other population (e.g. are *Betascan** outliers from EA1 outliers in any other population). All comparisons use Wilcoxon signed rank tests to compare outlier genes to all other genes within the same population. **Panel A**: *Betascan** outliers from EA1 do not have higher *Betascan** scores in any other population. **Panel B**: outliers in ISC1 have elevated scores in ISC2 (P =2 x 10^-4^) and marginally-significant elevation of scores in BM (P = 0.047). **Panel C**: outliers in BM show marginally-significant elevation of scores in EA1 and ISC2 (P = 0.05, P = 0.02). Only the ISC1-ISC2 enrichment passes a Bonferroni-corrected P-value threshold of 0.0125 (0.05/4).

# **Supplementary Tables**

**Supplementary Table 1.** List of isolates (note to reviewers: isolates sequenced in this study
have temporary sample accession numbers assigned by NCBI. We will update this before publication and when data have been released).

**Supplementary Table 2.** FST values for each population.

**Supplementary Table 3.** Genes that are *Betscan2* outliers in multiple populations.

**Supplementary Table 4.** Candidate gene set and justifications.

**Supplementary Table 5.** Nucleotide diversity and Tajima’s *D* values for all genes in all populations.

**Supplementary Table 6.** Annotated variants in candidate genes.
